# Mixed Results on the Efficacy of the CharacterMe Smartphone App to Improve Self-Control, Patience, and Emotional Regulation Competencies in Adolescents

**DOI:** 10.3389/fpsyg.2021.586713

**Published:** 2021-05-20

**Authors:** Sarah A. Schnitker, Jennifer Shubert, Juliette L. Ratchford, Matt Lumpkin, Benjamin J. Houltberg

**Affiliations:** ^1^Science of Virtues Laboratory, Department of Psychology and Neuroscience, Baylor University, Waco, TX, United States; ^2^Behavioral Science Department, Utah Valley University, Orem, UT, United States; ^3^Tidepool.org, Palo Alto, CA, United States; ^4^Search Institute, Minneapolis, MN, United States

**Keywords:** technology, intervention, development, character, patience, self-control, adolescence, emotion regulation

## Abstract

Unprecedented levels of access to adolescents' time and attention provide opportunities to convert traditional character and socioemotional competencies interventions into behavioral intervention technologies. However, these new tools must be evaluated rather than assuming previously validated activities will be efficacious when converted to a mobile platform. Thus, we sought to design and provide initial data on the effectiveness of the CharacterMe smartphone app to build self-control and patience, which are built on underlying social-emotional regulation competencies, in a sample of 618 adolescents (*M*_*age*_ = 16.07, Female = 56.6%). We also sought to examine whether framing the app activities as having a transcendent (spiritual connection or moral/prosocial) rather than instrumental purpose would increase engagement and change in self-control, patience, and emotion regulation. Finally, we tested the impact of framing activities as building strengths vs. fixing weaknesses. Results highlight the difficulty of translating psychological interventions to behavioral intervention technologies. Overall, the CharacterMe smartphone app was unsuccessful in increasing self-control, patience, or emotion regulation in adolescents, with analyses showing no significant mean changes over time. Framing conditions and user engagement were largely not significant predictors of change in self-control, patience, and emotion regulation.

## Introduction

Adolescents around the world have access to smartphones and spend significant amounts of time on their mobile devices. Rates of adolescents' smartphone access are at or above 95% for most developed nations and unrelated to parental income (Madden et al., 2013; Yong-Wan et al., 2017). Even among 21 developing countries, a median of 54% of adolescents per country own a smartphone or use the internet occasionally (Poushter, 2016). Moreover, adolescents who have a device use them frequently. In the USA, 45% of adolescents report “almost constantly” using their devices (Anderson and Jiang, 2018), and South Korean youth aged 10–19 report using their smartphones an average of 11 h per week (Yong-Wan et al., 2017).

Although numerous studies document the risks of high rates of various types of smartphone use among children and adolescents (e.g., problems related to addiction, anxiety, and depression; Jeong et al., 2016; Elhai et al., 2017), other research suggests adolescents may benefit when they engage with technology in healthy ways (e.g., Uhls et al., 2017; Moreno and Uhls, 2019). Given that smartphone use shows no signs of decreasing, it is imperative that researchers and designers work together to increase the ways adolescents can engage technology in a beneficial manner.

Given unprecedented levels of access to adolescents' time and attention through mobile devices, there is a unique opportunity for psychologists to convert traditional socioemotional learning and character strength interventions that promote positive youth development (e.g., Guerra and Bradshaw, 2008; Durlak et al., 2011; Proctor et al., 2011; Lavy, 2020) into behavioral intervention technologies. However, few such apps exist for youth. Numerous digital mental health interventions focus on ameliorating symptoms of mental illness and other physical health problems in children and adolescents (Liverpool et al., 2020; Temkin et al., 2020), but most of these do not focus on promoting more general socioemotional skills or character strengths in non-distressed populations. At the time of our app development, several scientifically vetted apps on the open marketplace delivered positive psychology interventions to adults. For example, the Happify app was a popular delivery tool for positive psychology, cognitive-behavioral therapy, and mindfulness-based activities and was soon validated in adults (Parks et al., 2018). However, few studies examined the effectiveness of similar apps among adolescent users. Since our data collection, a few standalone apps have reached the marketplace targeting self-regulatory capacities in adolescents (e.g., eScape by Hides et al., 2019; SmartCAT2.0 by Silk et al., 2020), but those were not available during our design process. Thus, we endeavored to assess whether digital media might be developed to cultivate character strengths in adolescents.

Although behavioral intervention technologies provide increased opportunity for user access and integration with daily life, they also exacerbate challenges related to user adherence and engagement that are less prominent for in-person interventions (Schueller et al., 2013). These challenges are especially pronounced when targeting adolescent users, who tend to have lower self-regulatory capacities (Blakemore and Choudhury, 2006; Opitz et al., 2012) and may require greater scaffolding to gain socioemotional and moral competencies that promote character strengths than adults (Schnitker et al., 2017). Thus, psychologists must not assume previously validated activities will be efficacious on a mobile platform for adolescents.

Given these opportunities and challenges, the aim of the present study was to design and conduct an initial assessment of the CharacterMe smartphone application's ability to build social-emotional capacities for emotion regulation (i.e., processes that monitor, assess, and modulate emotional reactions in goal pursuit; Zeman et al., 2002) that facilitate development of the character strengths of self-control (i.e., the ability to override predominant responses; Inzlicht et al., 2014) and patience (i.e., the ability to stay calm in the face of frustration, adversity, or suffering; Schnitker, 2012) among adolescents. We chose these strengths to offset broader concerns that smartphone use is associated with lower delay of gratification and impulse control (Wilmer and Chein, 2016) alongside findings that adolescents with lower self-regulatory abilities are more prone to the negative effects of smartphone use (e.g., addiction; Gökçearslan et al., 2016). Moreover, ethicists theorize self-control and patience assist in the acquisition and expression of a range of character strengths (Pianalto, 2016), and empirical data show these strengths promote positive social skills and well-being (Tangney et al., 2004; Schnitker, 2012; Ronen et al., 2016; Schnitker et al., 2017; Morrish et al., 2018; Lavelock et al., 2021). Researchers have successfully built apps to increase such regulatory strengths in adults; for example, Fishbach and Hofmann (2015) found a 1-week smartphone intervention increased self-control in the pursuit of personal goals. Thus, we were confident in the necessity and potential success of building a behavioral intervention technology for youth cultivating self-control and patience as well as the underlying social-emotional capacities for emotion-regulation.

Following an extensive design process, we developed an app around nine regulatory tasks. The first three tasks targeted the improvement of self-control based on the strength-energy model of self-control (Baumeister et al., 2007), which conceptualizes self-control as a domain-general strength that is depleted by short-term exertion but can increase through repeated use over time. At the time we developed our app, there were over a dozen studies that suggested enhancing domain specific self-control through small acts of practice translated to enhanced self-control in other domains for adults. Although a subsequent meta-analysis found that these self-control training effects were likely smaller and less stable than originally reported (Inzlicht and Berkman, 2015), there were still persistent effects for self-control practice in one domain increasing self-control across multiple domains. Researchers have begun testing such self-control interventions based on this model with adolescent samples; initial work suggests interventions can increase self-control and patience when not perceived as too difficult (Schnitker et al., 2017). Altogether, the effectiveness of self-control enhancing activities needs further exploration, but there is some empirical support for their potential with adolescents. Moreover, little is known about the impact of delivering these interventions through digital tools designed specifically for adolescents. Thus, *we hypothesized our three “Get Better” tasks grounded in the strength-energy model would increase the character strengths of self-control and patience as well as emotion regulation competencies in adolescents through a smartphone app (H1a)* because increases in domain general self-control would facilitate improvements for across all strengths and competencies requiring regulation. Task descriptions and the studies from which we adapted them are provided in Table 1.

**Table 1:** Description of CharacterMe tasks.

**Task**	**Participants instructed to…**	**Adapted from**
**Self-Control tasks**		
Hand swap	Use a non-dominant hand for 5–20 min	Schnitker et al., 2017
Math challenge	Solve math problems and then are interrupted with a chance to instead watch a video	Galla et al., 2014
Watch your mouth	Choose one word they say too much and avoid using it for 5–20 min	Gailliot et al., 2007
**Conflict resolution tasks**		
Listen up	Listen to a piece of music of their choice for 30 s that helps to improve mood	Moore, 2013
Mindfulness	Engage in a mindful breathing exercise for X min	Meiklejohn et al., 2012; Metz et al., 2013; Zoogman et al., 2015
Selfie	Take a picture depicting themselves during a conflict, then relax and breathe deeply for 60 s before taking another picture of themselves	Merry et al., 2004; Meiklejohn et al., 2012; Metz et al., 2013; Zoogman et al., 2015
Solutions	Brainstorm three ways to solve the conflict in a more positive manner then indicate the best solution and reflect on their choice	LeCroy and Rose, 1986; Johnson et al., 1997
Take perspective	Imagine how the other person felt during the conflict and rate the other person's emotions	LeCroy and Rose, 1986; Johnson et al., 1997; Beelmann and Heinemann, 2014
Think again	Reappraise their initial negative thoughts about the conflict	LeCroy and Rose, 1986; Merry et al., 2004; Schnitker, 2012

The remaining six tasks (also in Table 1) sought to cultivate patience in the context of interpersonal conflict by targeting underlying social-emotional competencies related to emotion regulation. Previous research suggests there are three types of patience that share a common core but distinctively express themselves for interpersonal frustrations, long-term life hardships, and short-term daily hassles (Schnitker, 2012). Adolescents encounter interpersonal stressors at a high rate (reporting an average of 7–8 conflicts per day; Laursen, 1995), so we focused on activities around engaging interpersonal conflicts in a more regulated and patient manner. Extant research demonstrates a connection between adolescents' abilities to resolve conflicts well and the self-regulatory skills we were interested in cultivating (e.g., Vera et al., 2004). Likewise, two previous studies with interventions specifically targeting increases in the virtue of patience among adults showed that activities involving meditation, cognitive reappraisal, emotional awareness, savoring the present, and adopting a positive viewpoint contributed to an increase in patience and well-being across time (Schnitker, 2012; Lavelock et al., 2021). Moreover, numerous studies testing specific strategies to build social-emotional competencies that underlie patience in adolescents support the efficacy of activities that build emotional recognition (Merry et al., 2004; Meiklejohn et al., 2012; Metz et al., 2013), use music to regulate mood (Moore, 2013), teach mindfulness meditation (Meiklejohn et al., 2012; Metz et al., 2013; Zoogman et al., 2015), generate potential interpersonal solutions for conflict (LeCroy and Rose, 1986; Johnson et al., 1997), facilitate perspective-taking (LeCroy and Rose, 1986; Johnson et al., 1997; Beelmann and Heinemann, 2014), and teach cognitive reappraisal/reframing (LeCroy and Rose, 1986; Merry et al., 2004; Schnitker, 2012). Although these tasks do not specifically target the development of self-control, they require self-control to engage, so we would expect that they might build self-control alongside the social-emotional competencies that underlie patience according to the strength-energy model. *Thus, we hypothesized that our six “Solve a Conflict” tasks would increase the character strengths of patience and self-control as well as emotion regulation competencies across time (H1b)*.

In order to examine the impact of technologically mediated interventions, it is important to consider the level of use and engagement by the target audience alongside the actual content of activities. Studies assessing positive psychology interventions and behavioral intervention technologies suggest intervention effectiveness is dependent on participant engagement and effort (Lyubomirsky et al., 2011; Schueller et al., 2013; Schnitker et al., 2017; Schnitker and Richardson, 2019). Designers can increase engagement by using small rewards (Thompson et al., 2008), enhancing user agency (Coyle et al., 2012), and highlighting an app's usefulness (Venkatesh and Davis, 2000). Accordingly, we designed the CharacterMe app to include an individual point-based participation reward system (without using *social* rewards that are more likely to activate addictive interaction), allow for activity choice, and include introductory videos explaining the potential positive effects of the activities. However, including these design elements does not guarantee high engagement, so attention to usage data is an essential component of the design process (Klasnja et al., 2011). We tested the hypothesis that *higher levels of engagement of particular tasks would predict changes in the character strengths of patience and self-control as well as emotion regulation competencies across time (H2)*.

Another important area to consider for building behavioral intervention technologies is the higher-level motivations that adolescents have for engaging in character building interventions (Schnitker et al., 2020). Therefore, we also examined whether framing the app as providing distinct types of benefits would change effectiveness and engagement. In their integrated framework for building behavioral intervention technologies for mental health, Mohr et al. (2014) argue designers need to theoretically address both the “why” and “how” of interventions to explicate the overarching goals alongside specific behavior change strategies. However, the aim to build self-regulatory skills could activate a wide variety of higher-order goals that might differentially affect the perceived usefulness of the app, and perceived usefulness predicts subsequent usage behavior (Venkatesh and Davis, 2000). Previous research with adolescents demonstrates that experimentally activating transcendent, or beyond-the-self, motives (e.g., helping others, making the world a better place) in contrast to self-oriented motives (e.g., getting rich, performing well to get a job) for educational activities increases academic self-control and grade point average (Yeager et al., 2014). We manipulated the framing of app activities through the introductory videos, a self-reflection task, and instructional language to emphasize the utility of the activities to either (a) foster spiritual purpose and connections, (b) promote moral development and prosociality, or (c) improve performance and success. *We hypothesized the first two framings with beyond-the-self components would lead to higher levels of app engagement and greater increases in the character strengths of patience and self-control as well as emotion regulation competencies across time (H3)*.

In addition to considering differential impact of providing self-oriented and beyond-the-self sources of motivation, it also important to consider the impact of strength-based vs. deficit-oriented approaches. We examined whether framings would differ based on how they were worded in terms of building strengths vs. fixing/preventing deficits (e.g., build connections vs. prevent disconnection). Positive psychologists claim that strengths-building interventions have beneficial effects beyond traditional deficit-repair approaches (e.g., Seligman et al., 2005), and research from the motivation literature shows that people are more successful in the pursuit of approach/promotion vs. avoidance/prevention goals (Elliot and Friedman, 2007). *We hypothesized that framing activities as strength building would lead to greater increases the character strengths of patience and self-control as well as emotion regulation competencies than framing activities as fixing deficits (H4)*.

In summary, the CharacterMe smartphone application was designed to engage adolescents in the use of research-informed strategies for building the character strengths of self-control and patience as well as underlying emotion regulation capacities. Specifically, we created three “Get Better” tasks based on the strength-energy model, which we hypothesized would increase self-control (*H1)*. We also targeted the development six emotion regulation strategies situated in interpersonal conflicts in our “Solve a Conflict” tasks, which we hypothesized would build patience and self-control, alongside the underlying social-emotional capacities for emotion regulation (*H2)*. We also sought to examine whether framing the app activities as having a transcendent (spiritual connection or moral/prosocial) rather than instrument purpose would increase engagement and change in self-control, patience, and emotion regulation (*H3*). Finally, we tested the impact of framing activities as building strengths vs. fixing weakness (*H4*). Following previous studies of character interventions in adolescents (e.g., Strengths Gym; Proctor et al., 2011), we sought to examine the effectiveness of the intervention across a 6-month period. Two meta-analyses of school-based programs developing many of the social-emotional competencies underlying our patience building activities found that positive changes in students persistent for at least 6 months after intervention (Durlak et al., 2011; Taylor et al., 2017). Thus, behavior intervention technologies converting in-person interventions to an online platform should seek similarly persistent effects.

## Materials and Methods

### App Design Process

Our team engaged in an iterative participatory design process (Spinuzzi, 2005) for building the smartphone app. We assembled an interdisciplinary team, which included a designer, personality/social psychologist, developmental psychologist, and developer. We sought to approach our collaboration with a focus practicing the virtues Steen (2013) deems essential for participatory design: cooperation, curiosity, creativity, empowerment, and reflexivity.

Our first step to design was conducting a literature review of the existing interventions that had been previously validated to cultivate character strengths. Next, we brainstormed ideas for app design. We also conducted a systematic assessment of the app marketplace to map the landscape of related products. We surveyed teens for their top five most-used apps as a way to determine which user interfaces and interactions would be conventional and expected by our target audience. We followed the convention of the simple, bottom navigation from Instagram and the bright, playful colors of Snapchat along with its whole-screen swiping gesture as a secondary navigation (see Supplementary Material for design process visuals).

One aspect common in apps popular among teens that we did not borrow was the deliberate use of *social* reward (e.g., posting to a social network and getting likes from friends) to create addictive patterns (e.g., Van Deursen et al., 2015) that lead to problematic smartphone use, which is consistently correlated with depressive and anxiety symptom severity (Elhai et al., 2017). We resolved early on to reject these patterns in our design because research suggests people low in self-regulation are more prone to smartphone addiction (Gökçearslan et al., 2016). Using social rewards that might promote addiction would undermine our efforts to increase self-regulation among our most vulnerable users. Thus, we designed a simple point-based system, assigning experience points to each activity completed for progression through 10 levels across the 2-week study period visible in an animated bar at the top of the home screen.

We (designer and researchers) continued forward to fully implementing our iterative participatory design process (Spinuzzi, 2005), which included a user survey and focus groups with adolescents at local high schools to gauge interest in various app activities and designs as well as conversations with stakeholders As we began to focus on patience, self-control, and emotion regulation as target strengths and capacities, we asked adolescents to describe real-life scenarios where they struggled in these areas and solicited feedback on how various technologically mediated tools might provide solutions for these needs. Adolescent focus group participants expressed that they would get caught in cycles of recurring interpersonal conflicts (though they used the terminology of “fights”) and would be very interested in tools that would help them try out new strategies for staying calm during or soon after the conflict. Thus, our adolescent focus groups indicated a “solve a conflict toolkit” was a highly attractive and intrinsically rewarding activity. They also expressed interest in “get better” activities that would feel more game-like and build regulatory skills.

Next, our designer mapped out data architecture and user experience in flow charts and sketches, which began describing the elements comprised in the behavioral intervention technology model (Mohr et al., 2014), answering why, what, how (conceptual and technical), and when questions. The designer and academics reconvened to tweak designs and then began the arduous process of working out the specific instructions for the tasks in the app. Whereas, the researchers on the team were biased toward material with high fidelity to the scientifically-tested intervention protocols, the designer and developer were biased toward pithiness, minimalism, and positive user experience. This friction mirrored Steen's (2011) identification of the tension of balancing the users' knowledge and ideas with the practitioners'/researchers' expertise inherent in human-centered design.

During this time, programming began in earnest that also attended to the back-end data structures and web interface that enabled researchers to edit framing conditions and access participant data. Finally, we began testing the design with users and continued to tweak the app. At this point, the app was approved in both the Apple and Google Play stores for download, but the study experience could only be accessed with a specific code provided by the research team. See also https://mattlumpkin.com/portfolio/characterme-2/ for more description of the design process.

### Sample and Procedure

Adolescents ranging in age from 15 to 19 (*N* = *618, M*_*age*_ = 16.07, *SD*_*age*_ = 0.99; Female = 56.6%) were recruited from nine high schools in metropolitan Los Angeles, CA after obtaining approval from the Institutional Review Board. Participants were ethnically diverse: 41.1% Asian, 29.5% Latinx, 12.9% White, 11.5% other, and 5.0% African American. Participants differed in self-reported socioeconomic status: 10.4% very poor or poor, 32.2% lower middle-class, 43.8% middle-class, 13.5% upper middle-class or rich.

Participants completed an online pretest (T1); engaged in the CharacterMe app for 2 weeks and completed an online post-test (T2); and completed online follow-up surveys at 1-month (T3) and 6-months (T4). We chose to require 2 weeks of app engagement based on previous character and emotion regulation interventions study durations in adolescents (e.g., Liehr and Diaz, 2010; Schnitker et al., 2017) and adults (e.g., Seligman et al., 2005; Mitchell et al., 2009; Fishbach and Hofmann, 2015) coupled with feasibility for participant retention and payment. Participants were compensated for their time through Amazon gift cards as follows: $14 for the T1 survey, $32 for app participation, $14 for the T2 survey, $20 for the T3 survey, and $20 for the T4 survey. Attrition rates were 30% at T2, 42% at T3, and 54% at T4. Although power analyses are not available for latent growth models, a sample size of at least 100 participants is preferable (Curran et al., 2010), suggesting the current sample is sufficiently powered. We report how we determined sample size and all data exclusions (if any) in the Supplementary Material.

Upon completing the first survey, participants were assigned to one of six framing conditions crossing the three purpose domains with the approach vs. avoidance orientation (spiritual approach, spiritual avoidance, moral approach, moral avoidance, instrumental approach, instrumental avoidance). They were instructed to watch two framing-specific videos related to the app, each highlighting the usefulness of the two types of tasks, and then complete a self-reflection exercise intended to help them internalize the app's manipulated usefulness to their own lives. See the Supplementary Material for links to the framing videos and reflection task instructions. The framing conditions were also reinforced through a second viewing of the two videos after ~1 week of app engagement.

Although the framing videos and reflection task were the primary mechanisms for manipulating the purpose of the app activities, we also intended to reinforce the framing through minor tweaks to the instructions for the Daily Thermometer, Hand Swap, and Watch Your Mouth activities in the app. There were no instructions specific to framings for any of the six conflict solving tasks. Due to researcher error, while engaging the back-end administrator interface, participants in four of the six conditions received the incorrect wording for the Thermometer, Hand Swap, and Watch Your Mouth tasks. Specifically, the instrumental/avoidance condition viewed moral/approach wording, the moral/avoidance condition viewed moral/approach wording, the moral/approach condition viewed spiritual/approach wording, and the spiritual/approach condition viewed the moral/approach wording. See a full accounting of the framing activities and instructions per condition in the App Framing Error Details page of the Supplementary Material. Although these instruction errors undermined the purity of the framing conditions, the manipulation was very subtle (i.e., replacement of a few words in introductory instruction screens; see Supplementary Table 1) in comparison to the much more explicit manipulation provided by the videos and engagement in the self-reflection exercise. Inspection of the reflection exercise responses showed that participants were applying the framings from the videos to their own lives. Thus, although the instruction errors within the app for three out of 10 activities undermined the purity of the conditions, there is still value in examining the framing results. However, all interpretations should be made with caution and not applied without replication.

The CharacterMe App was available for download in the Apple and Google Play stores or accessed through an online browser. A description of the design process and screenshots of the app are available in the Supplementary Material. Following participant assent and parental consent, participants were sent a code to allow access to the app. Participants had the option to enable push notifications, which provided daily reminders to engage in tasks (see Supplementary Material for schedule). Participants who were engaging with online browser version received reminder texts three times per week for 2 weeks.

Participants were asked to fill out a feeling thermometer task each day. They were given suggestions for basic self-control tasks and conflict resolution tasks to complete each day, but they were also allowed to choose their own tasks. For the conflict resolution tasks, adolescents detailed a recent conflict, indicated who was involved, and provided a reason for the conflict. Participants rated four emotions (anger, sadness, upset, and happiness) during the worst point of the conflict (e.g., “How angry were you?”) and following the task (e.g., “How do you feel now?”).

### Measures

The following measures were used in the present analyses. Items for these measures and descriptions of all other measures included in the study are listed in the Supplementary Material.

App engagement was assessed through the number of days participants engaged the app across the 14-day period, and by summing the number of times participants engaged in each specific task.

Patience was assessed with the 3-Factor Patience Questionnaire (Schnitker, 2012) for three factors: life hardships patience (e.g., “I am able to wait out tough times”), interpersonal patience (e.g., “I am patient with other people”), and daily hassles patience (e.g., “In general, waiting in lines does not bother me”). Responses ranged from 1 = *not like me at all* to 5 = *very much like me*. Previous research has demonstrated the utility of this scale to measure change in patience in response to short-term interventions among adolescents (Schnitker et al., 2017). Internal reliability omega coefficients were 0.53 for daily hassles, 0.64 for interpersonal, and 0.74 for life hardships patience.

Self-control was measured with the Brief Self-Control Scale (Tangney et al., 2004). In accordance with de Ridder et al. (2011), we assessed inhibitory self-control using items directed toward overcoming impulses (e.g., “I am good at resisting temptation”). Responses ranged from 1 = *not at all* to 5 = *very much*. Negatively worded items were reverse-scored to reflect higher inhibitory self-control. Numerous studies have used this scale to measure adolescent self-control across time and cultures (e.g., de Ridder et al., 2012; Li et al., 2019; Liang et al., 2020). Internal reliability was 0.53.

Emotion regulation was assessed with the Children's Emotion Management Scales (Zeman et al., 2002). Of interest were anger regulation (e.g., “When I'm feeling mad, I control my temper”) and sadness regulation (e.g., “When I am feeling sad, I control my crying and carry on”). Responses ranged from 0 = *not true* to 2 = *very true*. Numerous studies have used this scale to assess adolescent emotion regulation across time (e.g., McLaughlin et al., 2011), and scale findings correlate with respiratory sinus arrhythmia among adolescents (e.g., Cui et al., 2015). Internal reliability was 0.70 for anger regulation and 0.55 for sadness regulation.

### Analytic Plan

To examine the effectiveness of the app tasks in promoting anger and sadness regulation, self-control, and three factors of patience, latent growth curve models (LGCM) were estimated to examine within-person change across time. Unconditional LGCMs were estimated to examine overall change across time. Next, conditional LGCMs were estimated to include CharacterMe tasks, framings, and number of days of engagement with the app as predictors of change, controlling for age and gender^1^.

## Results

**Figure 1:**
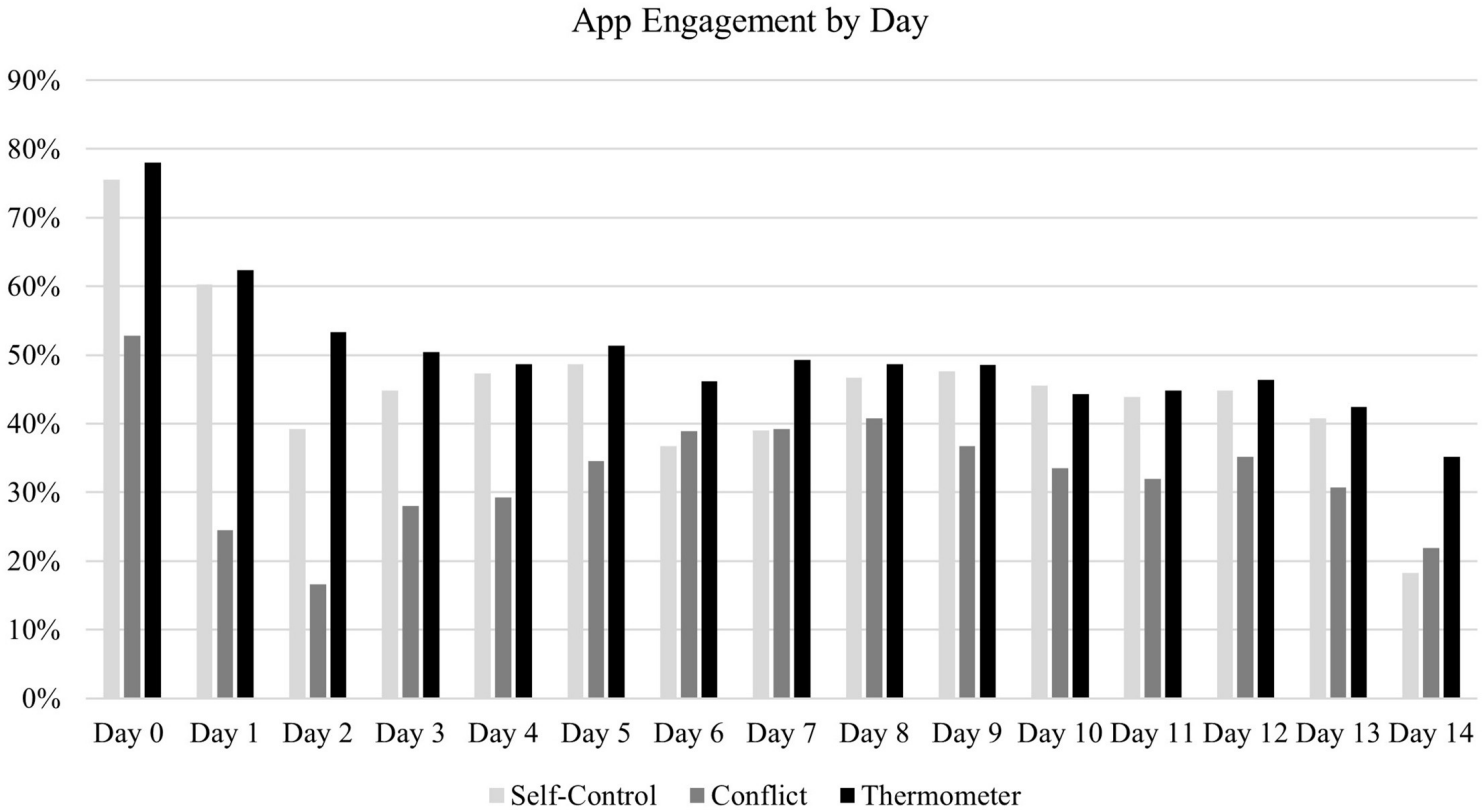
CharacterMe engagement across time.

App engagement across the course of the study is diplayed in Figure 1. Model fit indices for conditional and unconditional LGC models are shown in Table 2 and parameter estimates for LGC models are shown in Table 3.

**Table 2:** Model fit indices for latent growth curve models.

	**AIC**	**BIC**	**χ^2^(*df*)**	**CFI**	**TLI**	**RMSEA**
**Unconditional model**						
Life hardships patience	3928.84	3964.44	8.19 (6)	0.99	0.99	0.02
Interpersonal patience	3092.00	3132.05	21.44 (5)^***^	0.97	0.96	0.07
Daily hassles patience	3735.68	3771.28	20.67 (6)^**^	0.97	0.97	0.06
Self-Control	1923.68	1959.26	9.87 (6)	0.99	0.99	0.03
Anger regulation	2035.20	2075.24	10.31 (5)	0.98	0.98	0.04
Sadness regulation	1589.50	1625.10	21.43 (6)^**^	0.93	0.93	0.06
**Conditional model**						
Life hardships patience	3184.23	3297.42	67.51 (55)^***^	0.98	0.97	0.02
Interpersonal patience	2462.31	2575.50	82.71 (55)^**^	0.97	0.96	0.03
Daily hassles patience	3066.99	3180.18	96.48 (55)^***^	0.94	0.91	0.04
Self-Control	1513.85	1626.93	69.97 (55)	0.97	0.96	0.02
Anger regulation	1659.43	1776.82	61.57 (54)	0.98	0.97	0.02
Sadness regulation	1304.00	1417.19	83.37 (55)^**^	0.92	0.90	0.03

*AIC, Akaike Information Criteria; BIC, Bayesian Information Criterion; RMSEA, Root Mean Square Error of Approximation; CFI, Comparative Fit Index. For unconditional models of life hardships, daily hassles, and sadness regulation and for all conditional models except anger regulation, residuals for T4 values were small but negative, and thus fixed to 0*.

* *p < 0.05*,

** *p < 0.01*,

*** *p < 0.001*.

**Table 3:** Parameter estimates for LGC models.

	**Life hardships patience**	**Inter-personal patience**	**Daily hassles patience**	**Self-Control**	**Anger regulation**	**Sadness regulation**
**Unconditional model**						
*Mean*						
Intercept	3.27^***^	3.37^***^	3.38^***^	3.07^***^	2.13^***^	2.14^***^
Slope	0.01	0.00	0.00	−0.03	−0.00	−0.00
*Variance*						
Intercept	0.38^***^	0.34^***^	0.40^***^	0.12^***^	0.13^***^	0.07^***^
Slope	0.00^***^	0.00^***^	0.00^***^	0.00^***^	0.00	0.08^***^
**Conditional model**						
Gender	−0.009	−0.006	0.000	−0.001	−0.004	0.001
Age	−0.002	−0.001	−0.003	−0.002	−0.001	0.000
Hand swap	−0.001^***^	0.000	−0.001	0.000	0.000	0.000^*^
Math	−0.001	−0.001	0.000	−0.001	0.000	0.000
Watch your mouth	0.001^*^	0.000	0.000	0.000	0.000	0.000^*^
Listen up	−0.001	−0.001	−0.001	0.000	0.000	0.001
Mindfulness	−0.003	−0.004^*^	−0.002	0.000	−0.002	−0.002^*^
Selfie	0.002	0.000	−0.003	0.000	0.001	0.002
Solutions	−0.001	0.000	−0.001	0.000	0.000	0.001
Take perspective	0.002^***^	0.001^*^	0.001	0.000	0.000	−0.001^*^
Think again	−0.001	0.004^*^	0.002	−0.001	0.001	0.000
Total engagement	0.002^*^	0.000	0.000	0.000	−0.001	−0.002^**^
***Framing***						
Instrumental/Approach^a^	0.012	0.008	−0.010	0.000	0.010	0.005
Moral/Avoid^a^	0.030^**^	0.023^**^	0.001	−0.002	0.013	0.008
Moral/Approach^a^	0.019	0.006	0.004	−0.003	0.006	−0.001
Spiritual/Avoid^a^	−0.005	−0.002	−0.018	−0.013	−0.002	0.003
Spiritual/Approach^a^	0.004	0.016^*^	−0.012	0.002	0.009	0.004

a *Instrumental/Avoid framing is the reference*.

* *p < 0.05*,

** *p < 0.01*,

*** *p < 0.001*.

### Unconditional Models

For all six constructs, there were no significant changes over time, on average, which fails to provide evidence for *H1*. The variance of the slopes for sadness regulation (but not anger regulation), self-control, and all three types of patience were significant, suggesting interindividual differences in intraindividual change. Though significantly different from zero, the size of effects for variance in slope for self-control and the three patience factors were quite small (i.e., all coefficients <0.01); thus, any inferences based on effects related to variability in these variables should be made with extreme caution. In contrast, the variance of the slope for sadness regulation was similar in size to the variance of the intercept, which allows for solid grounding to make inferences about predictors of change in sadness regulation.

### Conditional Models

Participants who spent more days engaging with CharacterMe showed negative changes in sadness regulation but positive changes in life hardships patience. In other words, more time in the app was associated with decreases in sadness regulation and increases in life hardships patience across time.

Engagement with particular tasks was not a significant predictor of daily hassles patience, self-control, or anger regulation. Greater engagement with Hand Swap and Watch Your Mouth was positively associated with changes in sadness regulation, whereas greater engagement with Mindfulness and Take Perspective was negatively associated with within-person changes in sadness regulation. Changes in life hardships patience was positively associated with greater engagement with Take Perspective and Watch Your Mouth but negatively associated with Hand Swap. Engagement with Take Perspective and Think Again positively predicted within-person change in interpersonal patience whereas engagement with Mindfulness was negatively related. Thus, findings were mixed for *H2*.

There was limited support for *H3/H4*. Compared to instrumental/avoidance framings, moral/avoidance framings predicted more positive within-person change in life hardships and interpersonal patience whereas the spiritual/approach framing predicted positive change in interpersonal patience only.

## Discussion

Results highlight the difficulty of translating psychological interventions to behavioral intervention technologies. Overall, the CharacterMe smartphone app was unsuccessful in increasing self-control, patience, or emotion regulation in adolescents, with analyses showing no significant mean changes over time.

### Why Was the App Largely Ineffective?

#### User Engagement Provides Limited Information

Initial analysis suggests low levels of sustained user engagement may explain the app's ineffectiveness; by the 5th day, less than half of participants remained engaged (despite being paid for participation). However, low engagement does not seem to be the only reason for the null findings because the total days of engagement in the app was not a significant predictor of change for interpersonal patience, daily hassles patience, self-control, or anger regulation. Total engagement did predict very small increases in life hardship patience, but it also predicted very small decreases in sadness regulation. Though a decrease in sadness regulation was opposite the hypothesized effect, further analyses suggest it may reflect a willingness to accept sadness rather than suppress it.

Engagement with particular tasks only partially elucidates these findings further. Greater engagement in four of nine tasks (Hand Swap, Watch Your Mouth, Mindfulness, Take Perspective) led to small simultaneous increases and decreases in three out of six outcomes (life hardships patience, interpersonal patience, and sadness regulation). Though statistically significant, these effect sizes are extremely small, and there does not appear to be a meaningful pattern of findings that can be interpreted for life hardships or interpersonal patience. Thus, few conclusions should be made from these analyses.

The only pattern of effects for which we are willing to make tentative inferences are for the sadness regulation outcome, because it evinced greater variance for the slope than other measures. Higher levels of engagement in Hand Swap and Watch Your Mouth, which are built upon the strength-energy model of self-control, predicted increases in sadness regulation. In contrast, greater engagement with Mindfulness and Take Perspective tasks predicted decreases in sadness regulation. Although initially appearing contradictory, this pattern of effects makes sense upon further analysis. Hand Swap and Watch Your Mouth build inhibitory capacity, which would facilitate adolescents' abilities to suppress sadness. In contrast, the Mindfulness and Take Perspective tasks require adolescents to become more aware of their own and other's emotional states, which could solicit emotional reactivity that is difficult to regulate in adolescence (Cui et al., 2015), especially in relational contexts like interpersonal conflict. Additionally, the Mindfulness and Take Perspective tasks teach participants to experience emotions in a non-judgmental manner, which might increase their *acceptance* of sadness as an appropriate emotion under circumstances of loss such that they decrease efforts to suppress sadness (Liverant et al., 2008). This pattern of results could also suggest adolescents who experience heightened emotional reactivity from interpersonal conflicts chose to engage this task more often, but when coupled with the finding that total app engagement was associated with a similar decrease in sadness regulation, the interpretation that the mindfulness and perspective-taking tasks increase a willingness to accept sadness is more probable. Regardless, both enhanced reactivity and sadness acceptance may be appropriate responses after conflict that lead to relational repair or inhibition of relational aggression (Sullivan et al., 2010). However, they could also undermine the adolescent's ability to resolve conflict due to emotional hyperarousal and escalation (Moed et al., 2015). Future research is needed to explore this further as we did not assess subsequent relational repair attempts, relational aggression, or escalation.

#### Framings Only Partially Explain Low Efficacy

Researchers suggest establishing the usefulness of behavioral intervention technologies for users is essential for an app's success (Venkatesh and Davis, 2000), so another explanation for the null results might be that the app's utility was not evident to participants. We experimentally manipulated the purpose of the app by framing the activities as useful for (a) fostering spiritual purpose/connection, (b) promoting moral development, or (c) improving performance/success. Likewise, we influenced whether participants adopted an approach vs. avoidance motivation by experimentally manipulating the app's purpose as building strengths vs. fixing deficits. Overall, these framing conditions had minimal effects on outcomes. There were no significant differences based on framing condition for daily hassles patience, self-control, anger regulation, or sadness regulation.

There were significant but very small effects for interpersonal patience and life hardships patience. The spiritual/approach framing condition (i.e., receiving messages to “find purpose in belonging,” “find connection”) was associated with increases in interpersonal patience compared to the instrumental/avoidant framing group. This could suggest that the beyond-the-self motivation better promotes patience that is other- vs. self-directed. However, the effect did not extend to other outcomes and was so small as to make it insufficient evidence to support hypotheses 3 or 4, especially considering the error in framing instructions within the app for three activities. Future well-powered studies could explore whether such effects for beyond-the-self motivation replicate specifically for patience.

Similarly, participants in the moral/avoidance framing condition (i.e., receiving messages to “guard honor,” “defend character”) showed increases in both interpersonal and life hardships patience compared to the instrumental/avoidant framing group, but effects were very small and did not extend to other outcomes. Again, the errors in coding condition-specific instructions also limit inferences. We expected the moral framing to increase patience, but we did not hypothesize that the avoidant orientation would increase patience. Most research supports the benefits of approach motivations (Elliot and Friedman, 2007), but some studies suggest avoidant prosocial messages are more effective for people dispositionally-prone to avoidant motivation (Jeong et al., 2011). Although adolescents have some higher approach motivations than adults, they also report higher avoidance motivations (i.e., anxiety, fear; Gray et al., 2016), which might make them more sensitive to avoidant framing. However, we must reiterate that these effects were very small, and we found no differences for four of six outcomes. Thus, the overall conclusion must be that our results provided insufficient support for hypotheses related to the framings.

#### Other Potential Explanations and Future Directions

Given that differences in user engagement and manipulated purpose do not fully explain the null results for the study, we must consider other explanations for why the CharacterMe app was largely unsuccessful at increasing regulatory strengths. It is likely that intentionally growing self-control, patience, and emotion-regulation is a challenging activity for adolescents—so challenging that engagement in our app lacked the appropriate scaffolding to place activities within Vygotsky's zone of proximal development (Chaiklin, 2003). Previous studies have found that self-administered trainings to improve self-control, patience, and emotion regulation are only effective when perceived as not too difficult by adolescents, and some tasks, such as cognitive reappraisal exercises, may not be effective at all when self-administered (Schnitker et al., 2017). Our intent with the app was to scaffold the development of skills and strategies in daily life, but success may be dependent upon interpersonal engagement with a more highly skilled adult (Rhodes and Lowe, 2009) or peer (Tudge, 1992).

Future studies could utilize technology that includes direct scaffolding from a more highly skilled adult/peer. For instance, Silk et al. (2020) recently demonstrated promising results for the SmartCAT2.0 app to treat anxiety disorders among adolescents. Like CharacterMe, SmartCAT2.0 included similar exercises in sections of the app devoted to more general skill building and contextualizing skills in real-life situations. A key difference is that SmartCAT2.0 participants' therapists were able to use a clinician portal to monitor participant engagement within the app and discuss progress at in-person counseling sessions. Future studies using apps to build self-regulatory skills might test whether this in-person engagement is necessary for efficacy or whether interactions with skilled adults could be technologically mediated as well. Without such relational scaffolding, a more focused app involving limited activities might be more successful. For example, Hides et al. (2019) recently found that their Music eScape mobile app, which focuses exclusively on using music to manage emotions (akin to our Listen Up activity), improved emotion regulation in adolescents and emerging adults.

Although less likely, it is also possible that our study design is masking the positive effects of the app. We did not conduct a randomized controlled trial (RCT) whereby participants were assigned to a no-treatment control condition. It is possible that control participants would have decreased in patience, self-control, and emotion-regulation across the course of the study had they not used the app; so, it could be our app was successful in buffering normative declines. However, most research shows these capacities are stable or increasing (especially for girls) across adolescence (e.g., Branje et al., 2007). Our own data show the constructs were highly stable across time (i.e., no significant mean slopes for outcomes, very small effects for slope variance), so this explanation is highly unlikely. Future studies could employ RCT designs to eliminate this alternative explanation.

### Limitations and Lessons Learned

We measured self-control, patience, and emotion-regulation via adolescent self-reports, and several measures had low internal reliability despite previous use among adolescents. Alternate measurement modalities and instruments sensitive to developmental change might reveal additional significant results.

Second, we paid participants for their participation in the study, which may have inadvertently decreased engagement and effectiveness by undermining intrinsic rewards of the activities. However, the explicit endorsement of the app's utility through the framing conditions makes us believe that the participants were not unaware of the potential benefits, which previous work shows increases engagement (Venkatesh and Davis, 2000).

Finally, we were disappointed that our own errors produced during engagement with the app's back-end administrator interface, which limited the certainty with which we can make inferences from the findings related to the framing conditions. Although we were in conversations with our designer about the ideal administrator interface throughout the process, we obviously overestimated the capabilities of the research team to learn a new interface quickly and sufficiently enough to set up the study without error. We hope our mistake will push other teams of designers, developers, and researchers engaged in similar projects to design the back-end administrator interface with more checks and balances and simplicity to safeguard against researcher error or incompetence.

### Conclusion

Finding some small positive effects of the CharacterMe app as well as other apps new to the marketplace offer promise that researchers and designers can leverage digital media to promote character strengths like self-control and patience alongside emotion regulation capacities in adolescents. However, null and negative effects underscore the importance of scientifically assessing apps before releasing them on the marketplace—even when they deploy scientifically-vetted activities.

## Data Availability Statement

The datasets presented in this study can be found in the Open Science Framework online repositories at https://osf.io/jezf6/.

## Ethics Statement

The studies involving human participants were reviewed and approved by Fuller Theological Seminary School of Psychology Human Subjects Research Committee. Written informed consent to participate in this study was provided by the participants' legal guardian/next of kin.

## Author Contributions

SS conceptualized app and study design, managed data collection, wrote the introduction and discussion, and made revisions after review. JS conducted data analyses and wrote the results. JR wrote the method, assisted in study design, collected data, and ran supplementary analyses. ML designed the app. BH conceptualized app and study design, managed data collection, and revised the paper. All authors contributed to the article and approved the submitted version.

## Conflict of Interest

The authors declare that the research was conducted in the absence of any commercial or financial relationships that could be construed as a potential conflict of interest.

## Footnotes

^1^Additionally, we ran exploratory supplemental analyses, which included religiosity predictors as covariates in the conditional LGCMs. These findings are reported in the Supplemental Material.

## Supplementary Material

Supplementary materials include a description of the design process, additional sample size and data exclusion information, framing condition materials, screen captures of the CharacterMe app, push notification schedule for participants, measure items for scales analyzed, description of all study measures, app framing error details, and supplemental analyses.
