# Supplementary material for: Mixed Results on the Efficacy of the CharacterMe Smartphone App to Improve Self-Control, Patience, and Emotional Regulation Competencies in Adolescents
Source: Front Psychol. 2021 May 20;12:586713. doi: 10.3389/fpsyg.2021.586713 (PMC8173072; doi:10.3389/fpsyg.2021.586713)
Supplement: Supplementary file 1 [file Data_Sheet_1.PDF]

Online Supplement Materials

Mixed Results on the Efficacy of the CharacterMe Smartphone App to Improve Self-Control, Patience, and Emotional Regulation Competencies in Adolescents

Sarah A. Schnitker[1*], Jennifer Shubert[2], Juliette L. Ratchford[1], Matt Lumpkin[3], Benjamin J. Houltberg[4]

[1]Science of Virtues Laboratory, Department of Psychology and Neuroscience, Baylor University, Waco, TX, USA

[2]Behavioral Science Department, Utah Valley University, Orem, UT, USA

[3]Product Designer, Tidepool.org, Palo Alto, CA, USA

[4]Search Institute, Minneapolis, MN, USA

*** Correspondence:**
Corresponding Author
sarah_schnitker@baylor.edu

Table of Contents

	Page
Design Process Visuals	2
Sample Size and Data Exclusions	4
Framing Condition Materials	
Links to videos for framings	5
Self-reflection activity with videos	6
Screen Captures of CharacterMe App	8
Push Notification Schedule for CharacterMe Participants	15
Measure Items for Scales in Analyses	16
Description of All Study Measures	19
App Framing Error Details	25
Supplemental Table 1. Condition specific framing language	26
Supplemental Analyses with religiosity as covariate	27
Supplemental Table 2. Parameter estimates from LGC models controlling for religiosity	28

Design Process

See also https://mattlumpkin.com/portfolio/characterme-2/ for more description of the design process.

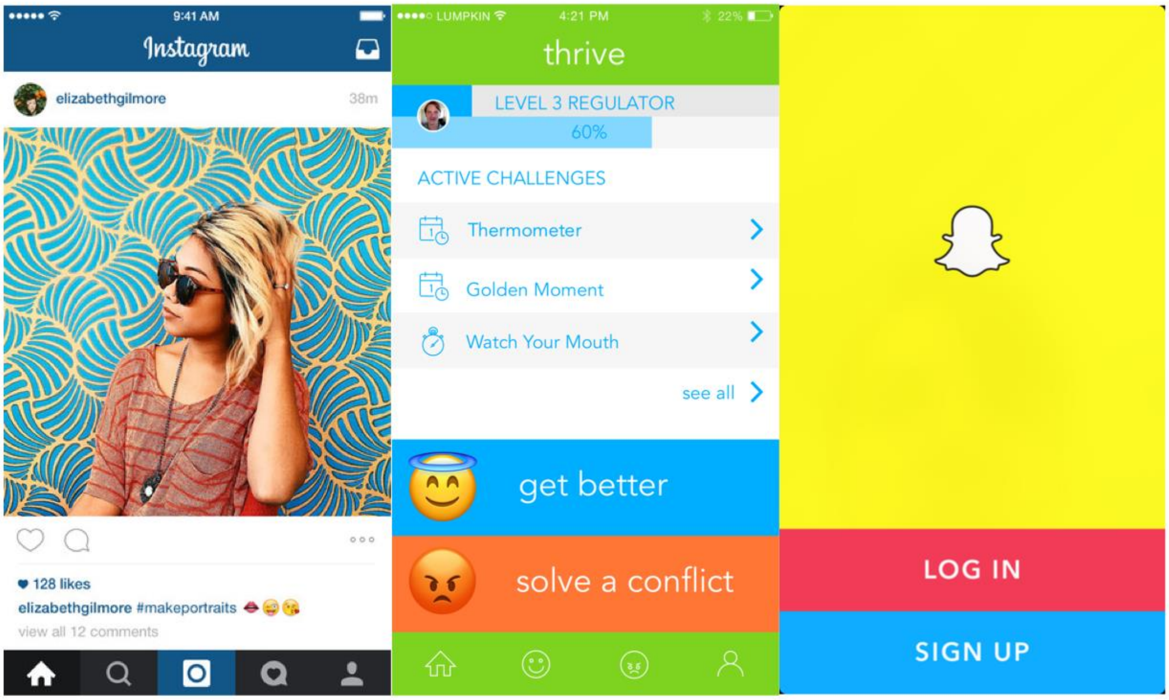

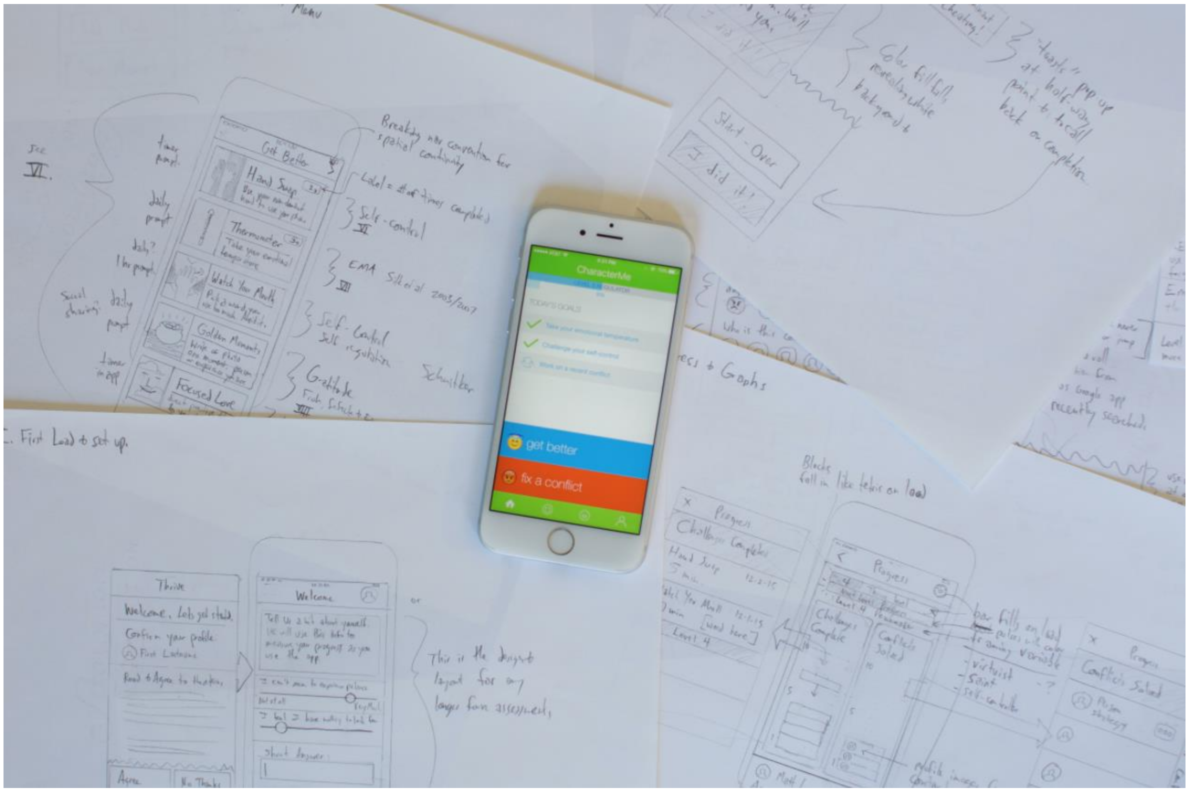

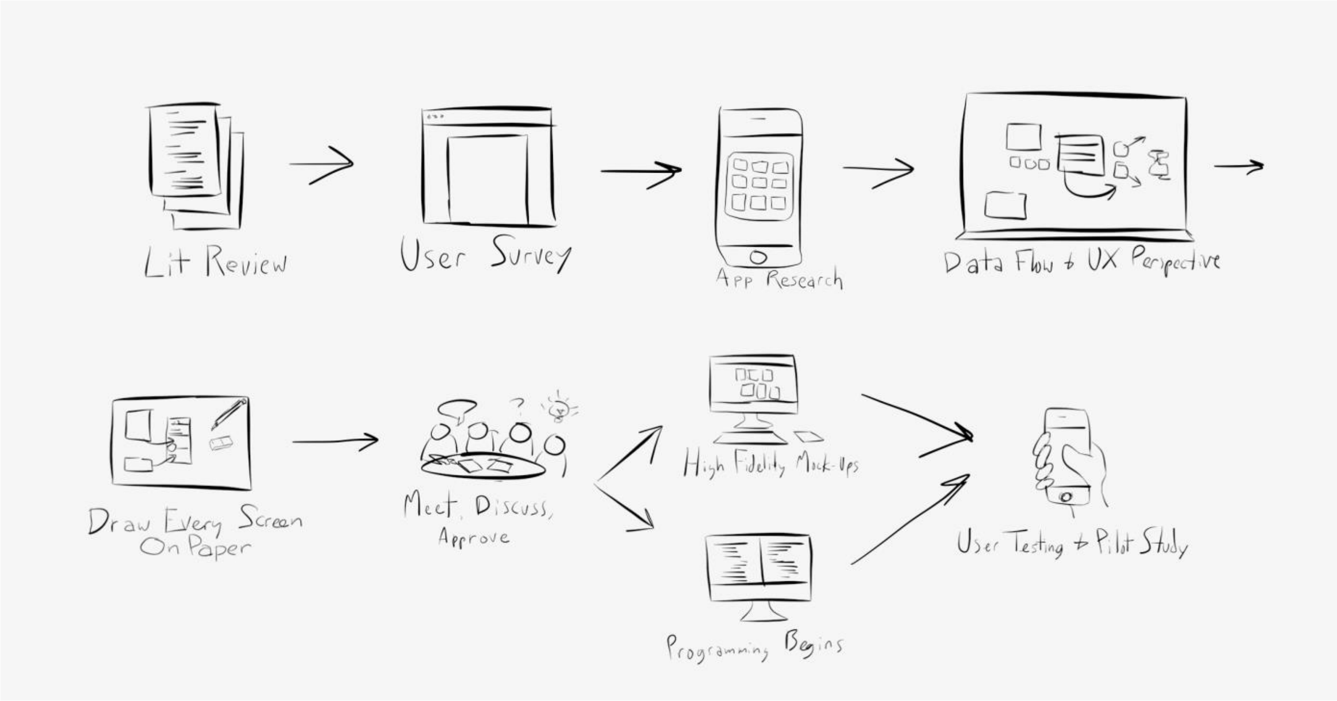
Lit Review
User Survey
App Research
Data Flow + UX Perspective
Draw Every Screen On Paper
Meet, Discuss, Approve
High Fidelity Mock-Ups
Programming Begins
User Testing + Pilot Study

Sample Size and Data Exclusions

Sample Size

Original a priori calculations from G*Power indicated that to test for six conditions with moderate effects (Cohen's d = .28-.56 based on Yeager et al., 2014 study of transcendent framing) 88 participants were necessary for 95% power (calculated as independent samples t-tests with d = .50). For omnibus ANOVA for six groups with moderate effects (f = .25), a total sample of 324 participants were required with 54 per condition. Given the difficulties of recruiting participants in this setting, lack of clear a priori effect sizes, and potential attrition, we recruited as many participants as possible among high schools and teams where we had access.

Power

Although power analyses are not available for latent growth models run in this study, a sample size of at least 100 participants is preferable (Curran et al., 2010), suggesting the current sample is sufficiently powered.

Data Exclusions

No data were excluded from the dataset. Participants who did not have any responses for independent variables in the present study (n = 156) were not included in analyses.

Framing Video Links
Get Better Instrumental Approach
https://youtu.be/L1mXh4lKFRA

Solve Conflict Instrumental Approach
https://youtu.be/_RaODu_z-Yg

Get Better Instrumental Avoidance
https://youtu.be/59ii9QrQbfY

Solve Conflict Instrumental Avoidance
https://youtu.be/ov0I7o4-a2w

Get Better Spiritual Approach
https://youtu.be/PunI4aoBzSE

Solve Conflict Spiritual Approach
https://youtu.be/qds1vpuS2qo

Get Better Spiritual Avoidance
https://youtu.be/Q_CgoRBDIvI

Solve Conflict Spiritual Avoidance
https://youtu.be/l2EX4UeaSg4

Get Better Moral Approach
https://youtu.be/UZ-Znyy9Tg4

Solve Conflict Moral Approach
https://youtu.be/WdkiGCyWsDo

Get Better Moral Avoidance
https://youtu.be/8ACFK4vNhKM

Solve Conflict Moral Avoidance
https://youtu.be/WdkiGCyWsDo

Spiritual Framings –Overcoming conflict and Get better – Approach

Alexis, the runner in these videos, has found that sports are a great way to connect to other people and to something greater than just herself. Even though doing her best is still important, Alexis has found that there is more to sports than just winning. Alexis has discovered a sense of purpose in belonging to a team where she feels supported and has opportunities to help those around her. This connection inspires her to find other areas in life where she experiences fulfillment in thinking about others and contributing to something that is bigger than herself.

How about you? Think about a sport, extracurricular activity, or hobby that is really important to you. How can striving for excellence in this activity help you to connect with something bigger than yourself? How can working hard at this activity create purpose and passion in your life? How can all your effort help you to give back some day?

Please take the next 7 minutes to write about these questions. Do not worry about spelling or grammar. Just write what you are thinking and feeling.

Spiritual Framings –Overcoming conflict and Get better – Avoidance

Alexis, the runner in these videos, has found that sports are a great way to prevent becoming disconnected from others and focused too much on herself. Even though doing her best is still important, Alexis has found that there is more to sports than just winning. She has discovered that belonging to a team keeps her from feeling unsupported or incapable of helping others. In order to avoid feeling unfulfilled, Alexis knows she needs to make sure she doesn't fail to find other areas in life to think about others and do something beyond herself.

How about you? Think about a sport, extracurricular activity, or hobby that is really important to you. How can striving for excellence in this activity help you to avoid disconnection and isolation? How can working hard at this activity help you avoid a sense of emptiness in your life and keep from missing your purpose? How can all your effort ensure you can give back some day?

Please take the next 7 minutes to write about these questions. Do not worry about spelling or grammar. Just write what you are thinking and feeling.

Moral Framings –Overcoming conflict and Get better – Approach

Alexis, the runner in these videos, has found that sports can give her an opportunity to be a better person and citizen. Even though doing her best is still important, Alexis has found that there is more to sports than just winning. Alexis believes that being a good teammate is the right thing to do and that her character is more important than how she performs. Alexis strives to be a good person outside of sports as well.

How about you? Think about a sport, extracurricular activity, or hobby that is really important to you. How can striving for excellence in this activity help you to become a good teammate and person? How can working hard at this activity make you more loyal, fair, and just? How can all your effort help you to build your character?

Please take the next 7 minutes to write about these questions. Do not worry about spelling or grammar. Just write what you are thinking and feeling.

Moral Framings –Overcoming conflict and Get better – Avoidance

Alexis, the runner in these videos, has found that sports prevent her from becoming a bad person and teammate. Even though doing her best is still important, Alexis has found that there is more to sports than just winning. Alexis believes that failing to fulfill her duty as teammate is wrong and that defending her character is more important than how she performs. Alexis strives to guard her honor outside of sports as well.

How about you? Think about a sport, extracurricular activity, or hobby that is really important to you. How can striving for excellence in this activity help you keep from becoming a bad teammate or bad person? How can working hard at this activity help you avoid disloyalty, unfairness, and injustice in your life? How can all your effort help you to defend your character?

Please take the next 7 minutes to write about these questions. Do not worry about spelling or grammar. Just write what you are thinking and feeling.

Instrumental Framings –Overcoming conflict and Get better – Approach

Alexis, the runner in these videos, has found that sports are a great way to push herself to the limits in order to be the best. She works very hard everyday so that she can be prepared to win in competition. Alexis sees the importance of working with her teammates in order to make each other better. She has discovered the importance of dedication and focus in order to achieve her goals and feel good about herself. Alexis knows that this will help her to perform better in many different areas of life.

How about you? Think about a sport, extracurricular activity, or hobby that is really important to you. How can striving for excellence in this activity help you to win in competitions or be the best at your activity? How can working hard at this activity make you feel good about yourself and your accomplishments? How can all your effort help you to perform better in other areas of your life?

Please take the next 7 minutes to write about these questions. Do not worry about spelling or grammar. Just write what you are thinking and feeling.

Instrumental Framings –Overcoming conflict and Get better – Avoidance

Alexis, the runner in these videos, has found that sports can keep her from being lazy and a loser. She feels if she does not work hard everyday, she will not be able to win. Alexis sees how not working with her teammates can prevent her from getting better in sports. She believes that a lack of dedication and focus will keep her from achieving goals and then she will not feel good about herself. Alexis knows that hard work in sports will prevent poor performance in many different areas of life.

How about you? Think about a sport, extracurricular activity, or hobby that is really important to you. How can striving for excellence in this activity help you to keep from losing in competitions or be the worst at your activity? How can working hard at this activity prevent you from feeling bad about yourself and your accomplishments? How can all your effort help you to avoid failure in other areas of your life?

Please take the next 7 minutes to write about these questions. Do not worry about spelling or grammar. Just write what you are thinking and feeling.

CharacterMe Main Screens

The home screen features a progress summary at the top and a list of the day's goals. As each goal is completed, it is checked off the list and the progress summary is updated.

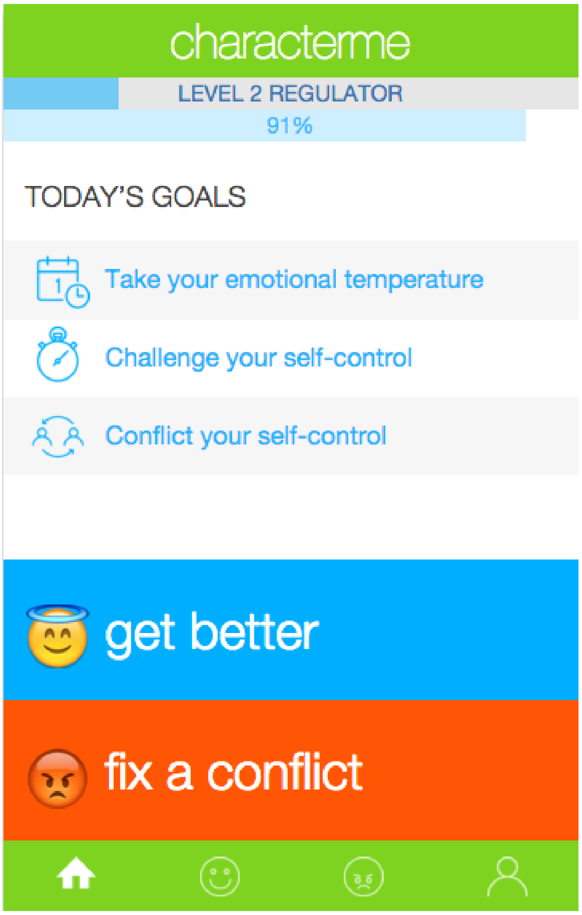

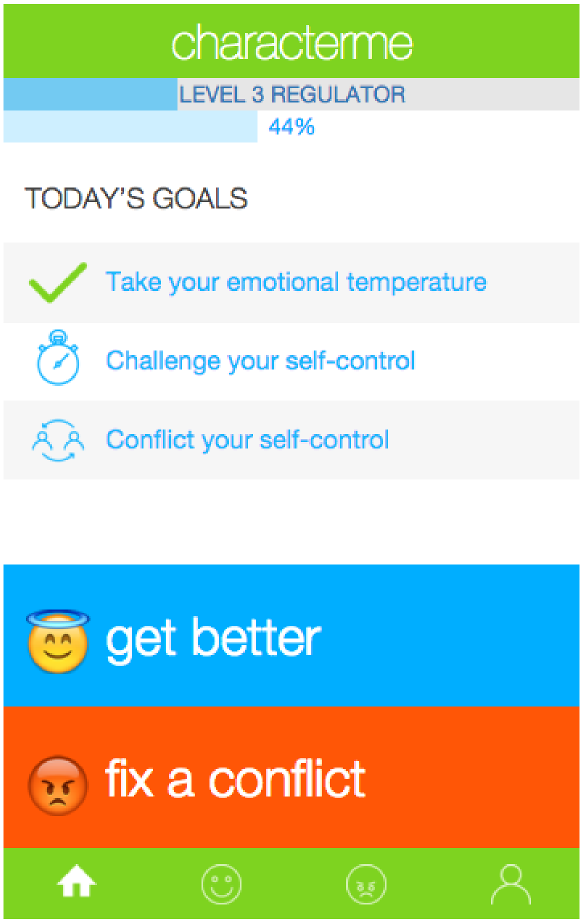

The “get better” and “fix a conflict” menus offer several challenges and strategies to improve self-control and resolve conflict. More options are unlocked as personal app activity increases.

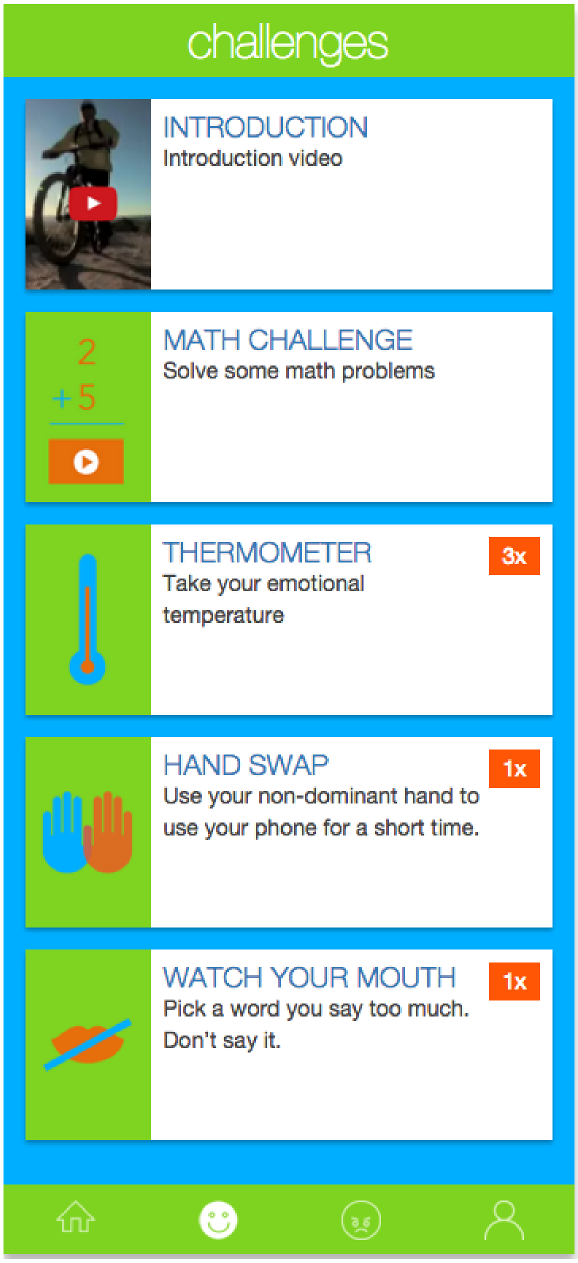

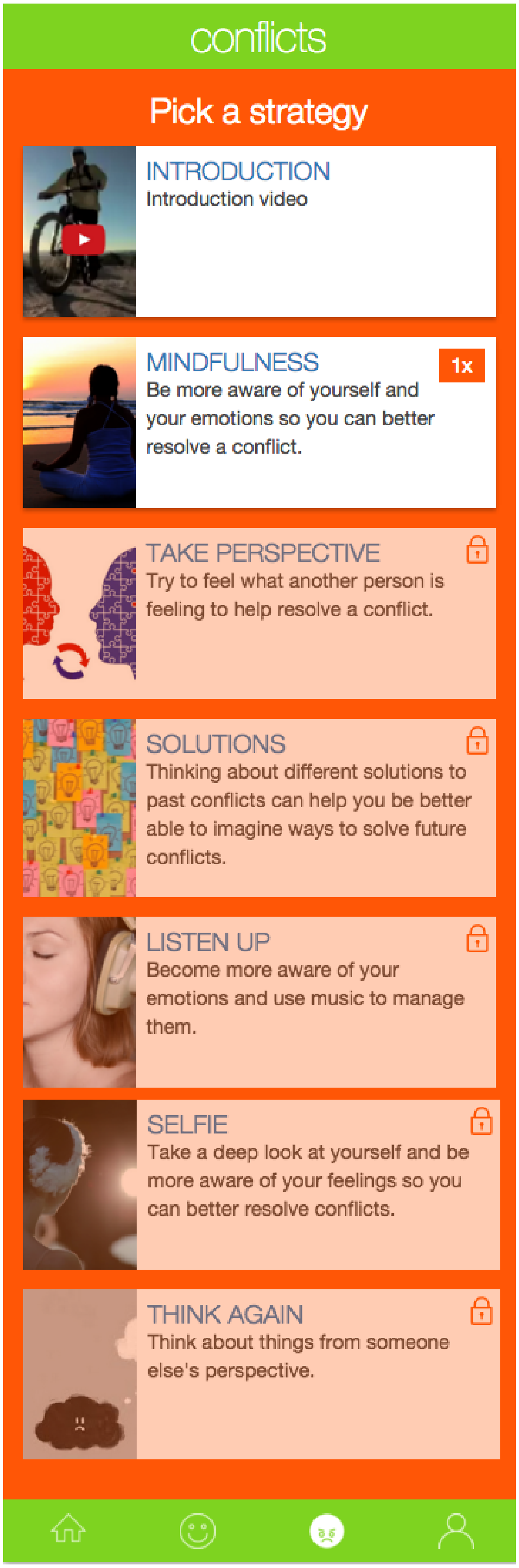

On the personal progress menu, users can review their current progress level and what activities they have completed. A settings menu allows the user to control their notification preferences.

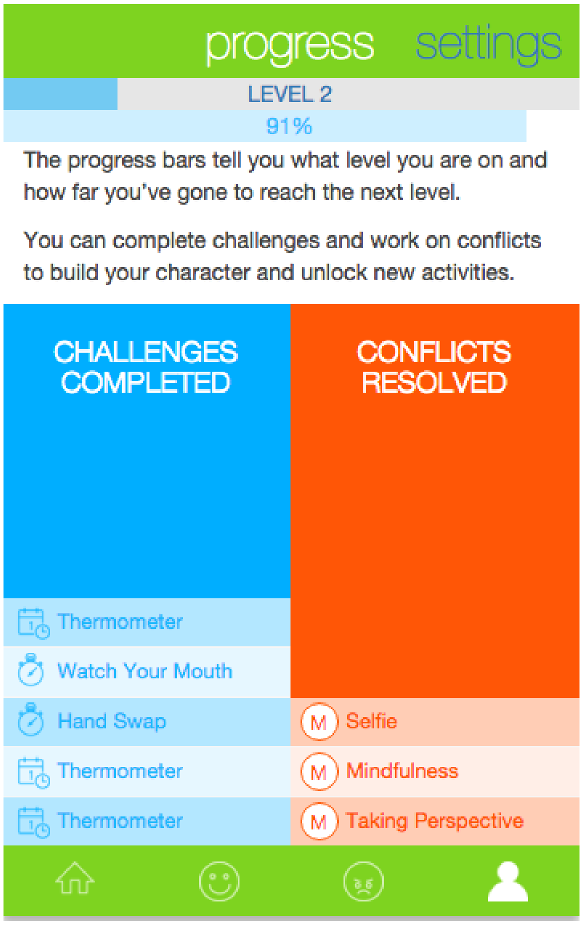

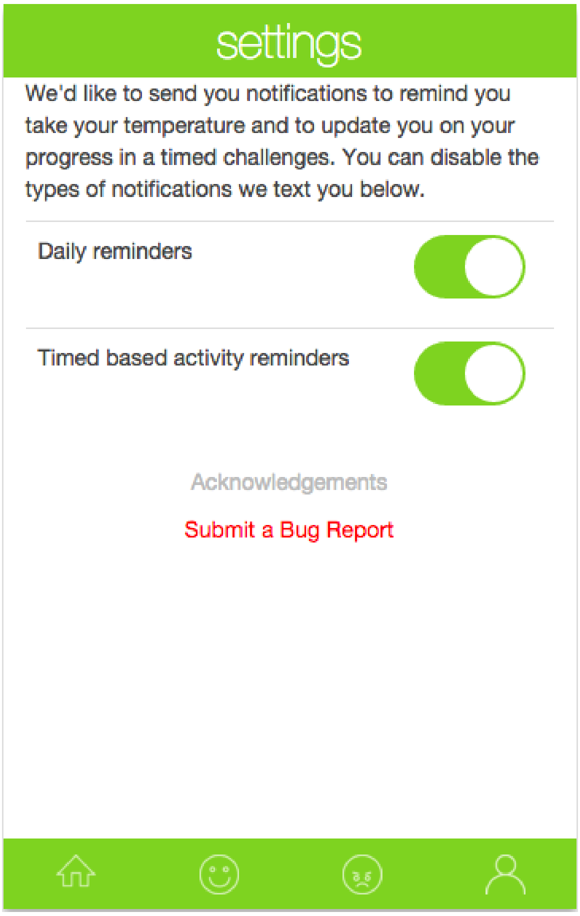

Get Better Challenge: Thermometer

The “Thermometer” is a daily challenge that prompts the user to take their emotional temperature at random points in time throughout the day. The user reflects on how they currently feel, how they felt in the past hour, and what they did about how they felt.

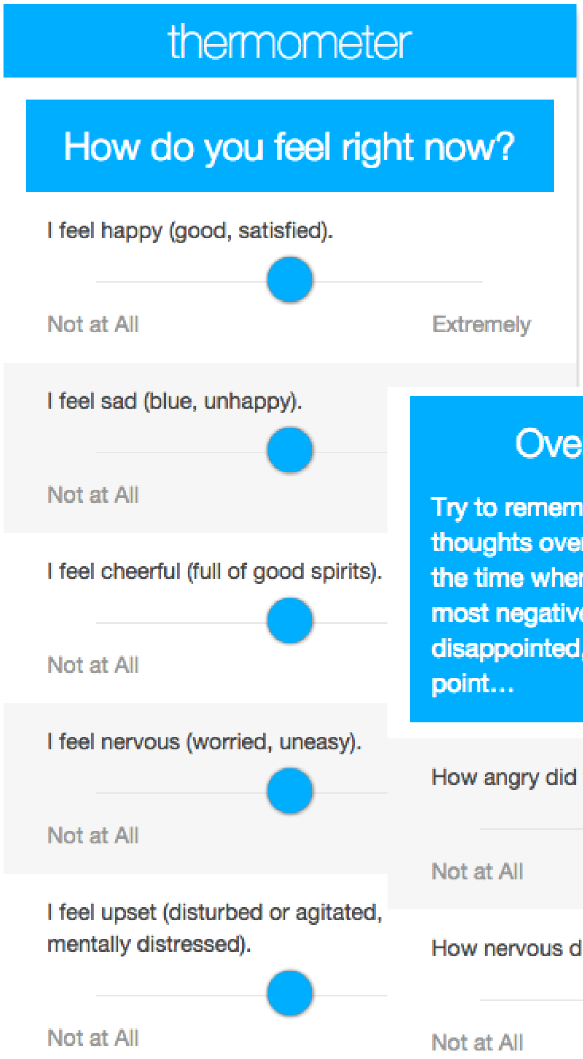

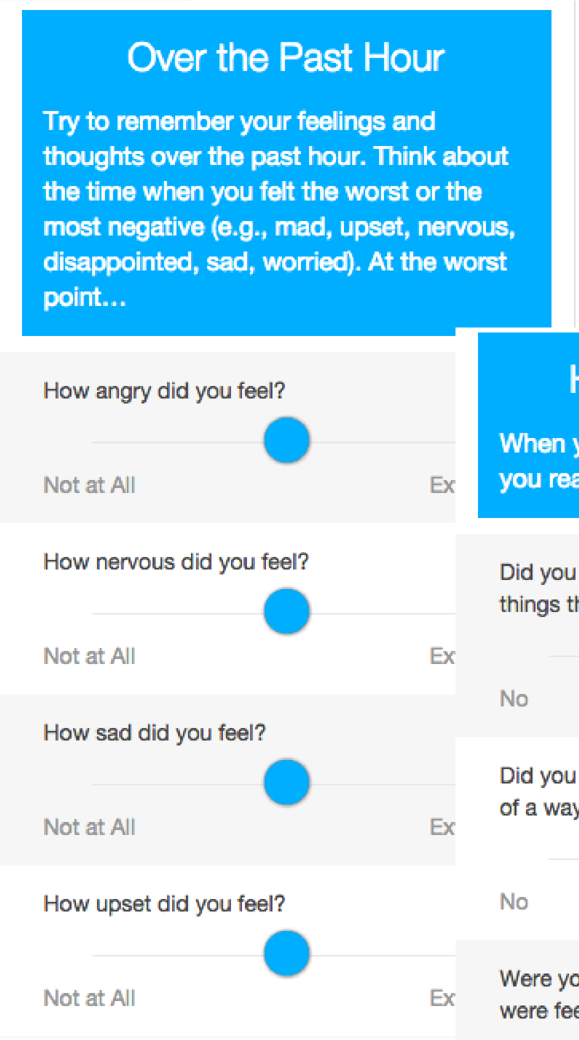

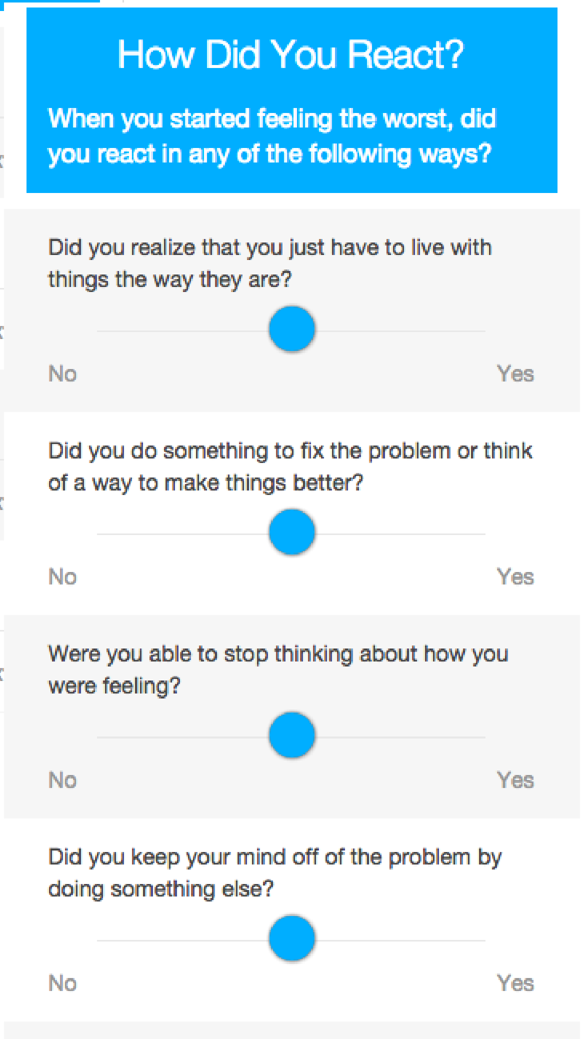

Get Better Challenge: Watch Your Mouth

In "Watch Your Mouth," the user is challenged to pick a word they say too much and to choose an amount of time to avoid saying that word.

A timer screen helps them see how long they have until the challenge is complete.

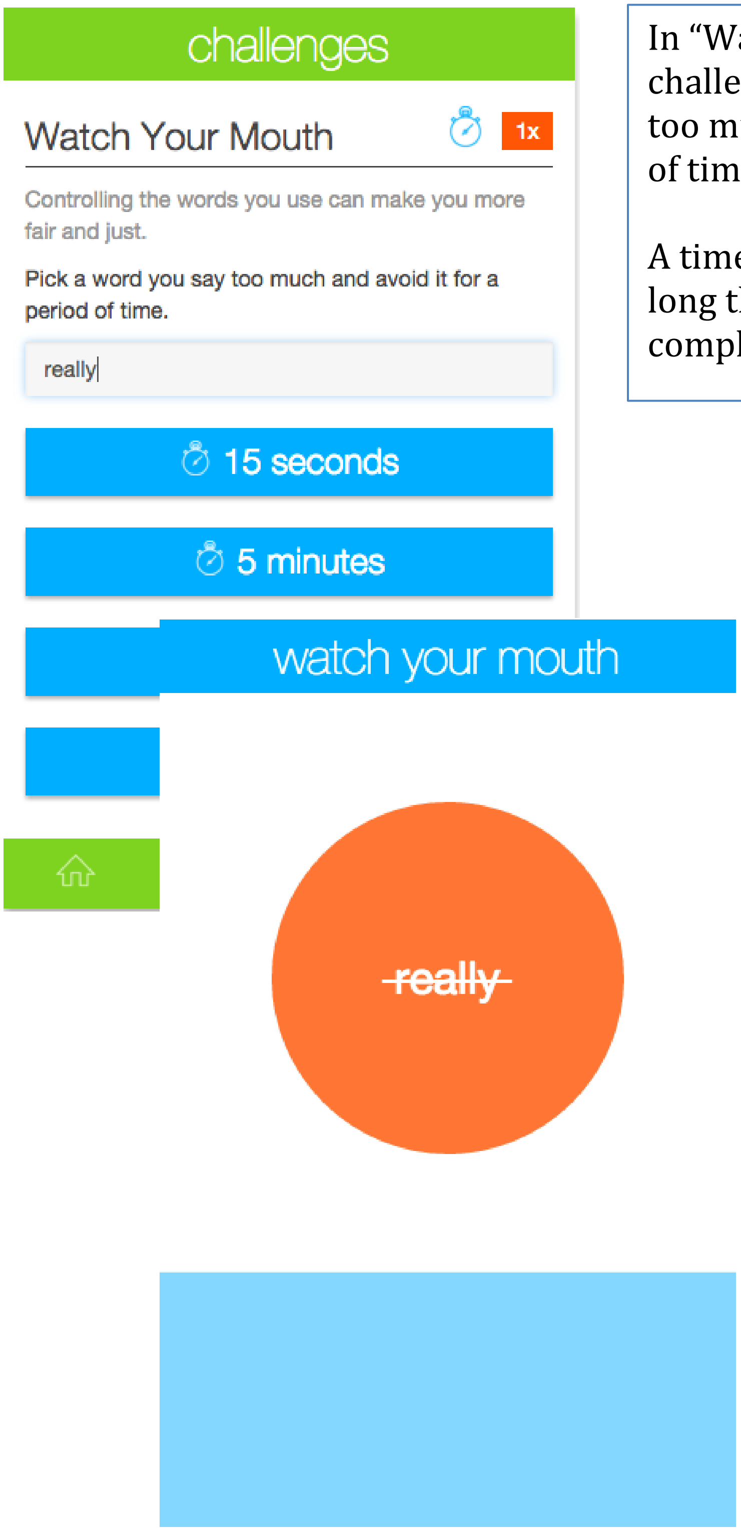

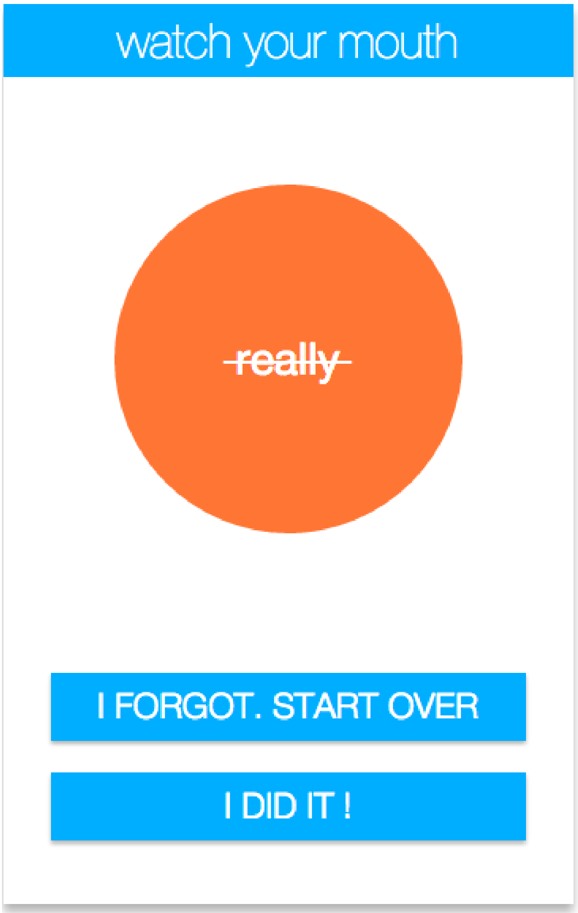

Fix a Conflict: Solutions

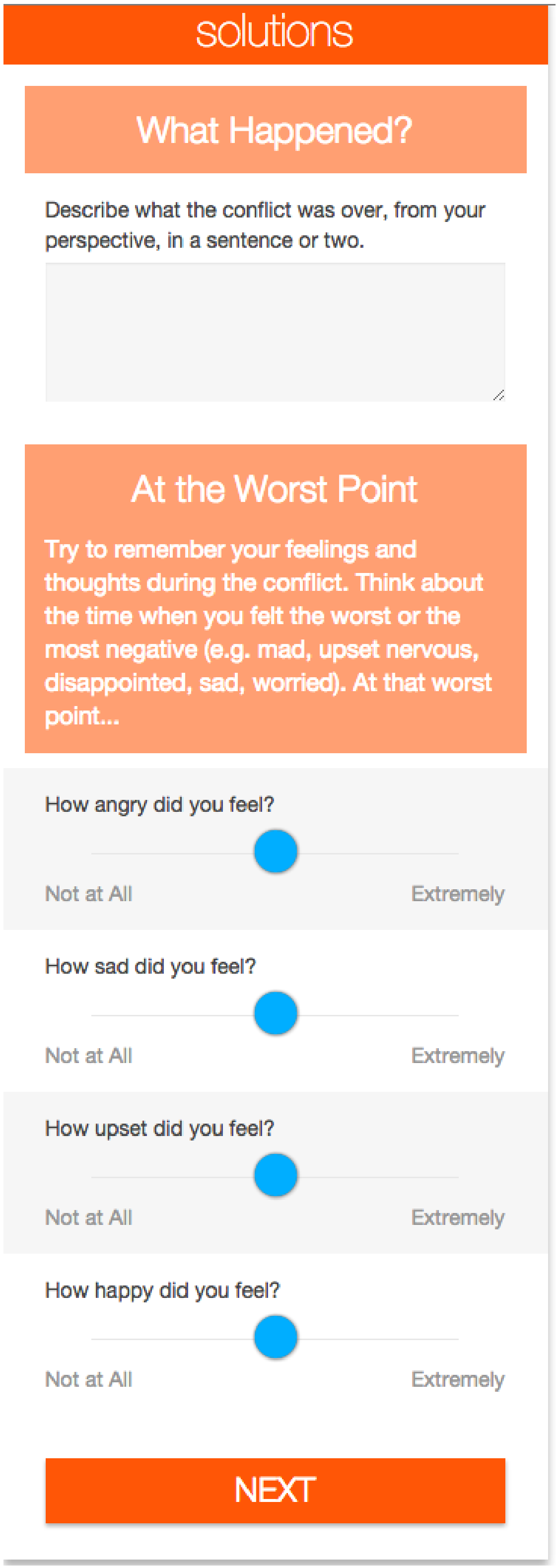

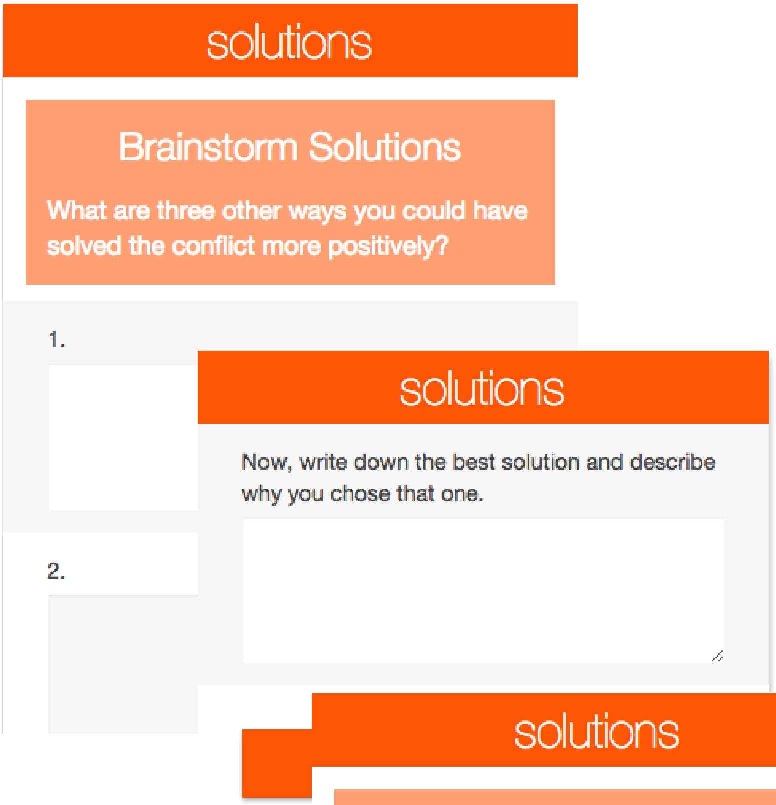

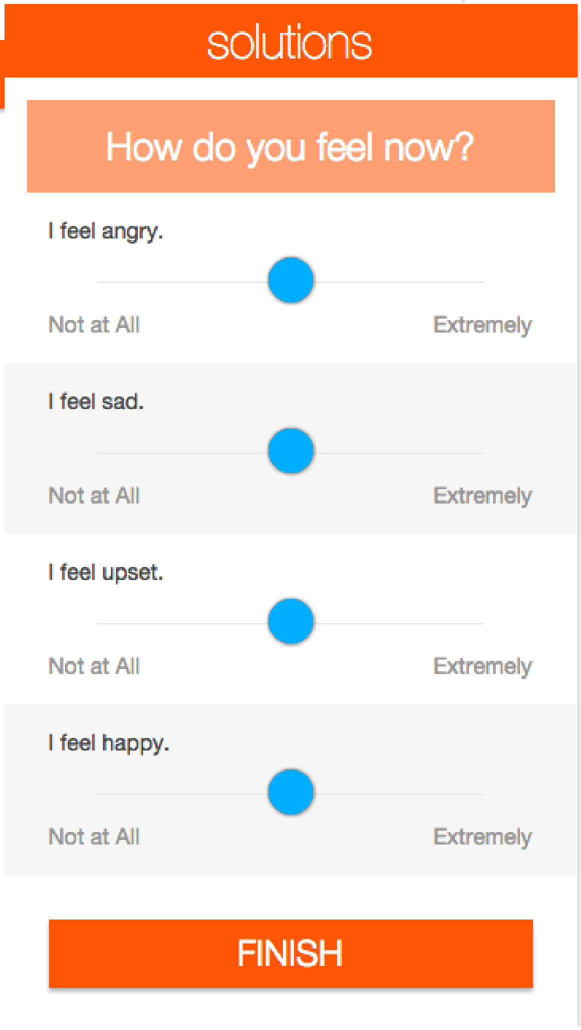

Each "Fix a Conflict" strategy begins with an opportunity to reflect on a conflict and how the user felt during the conflict and how they reacted. The "Solutions" strategy guides the user to think through other ways they could have solved the conflict that could benefit both people in the situation.

Fix a Conflict: Listen Up

In "Listen Up," after the user reflects on their current conflict, they are prompted to spend some time listening to music that will improve their mood. Afterward, they reflect on how they feel.

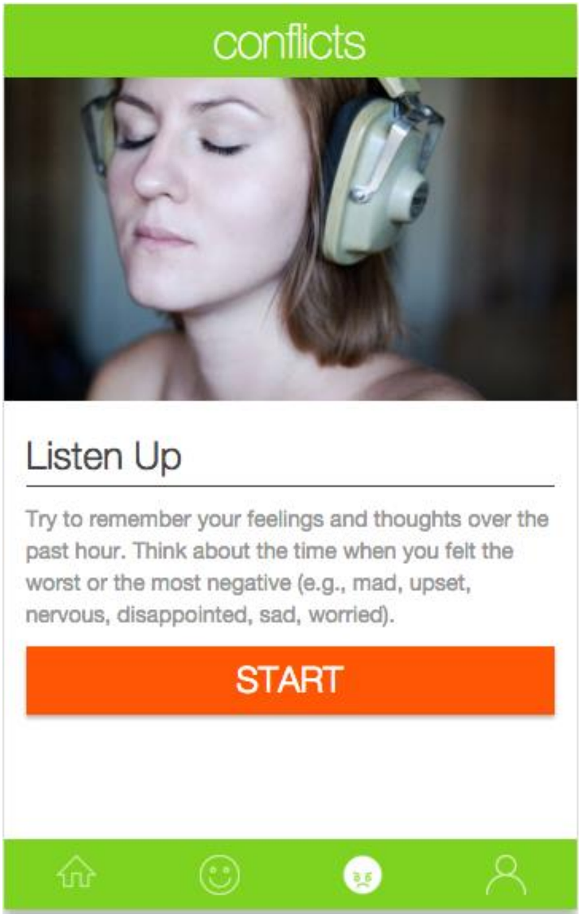

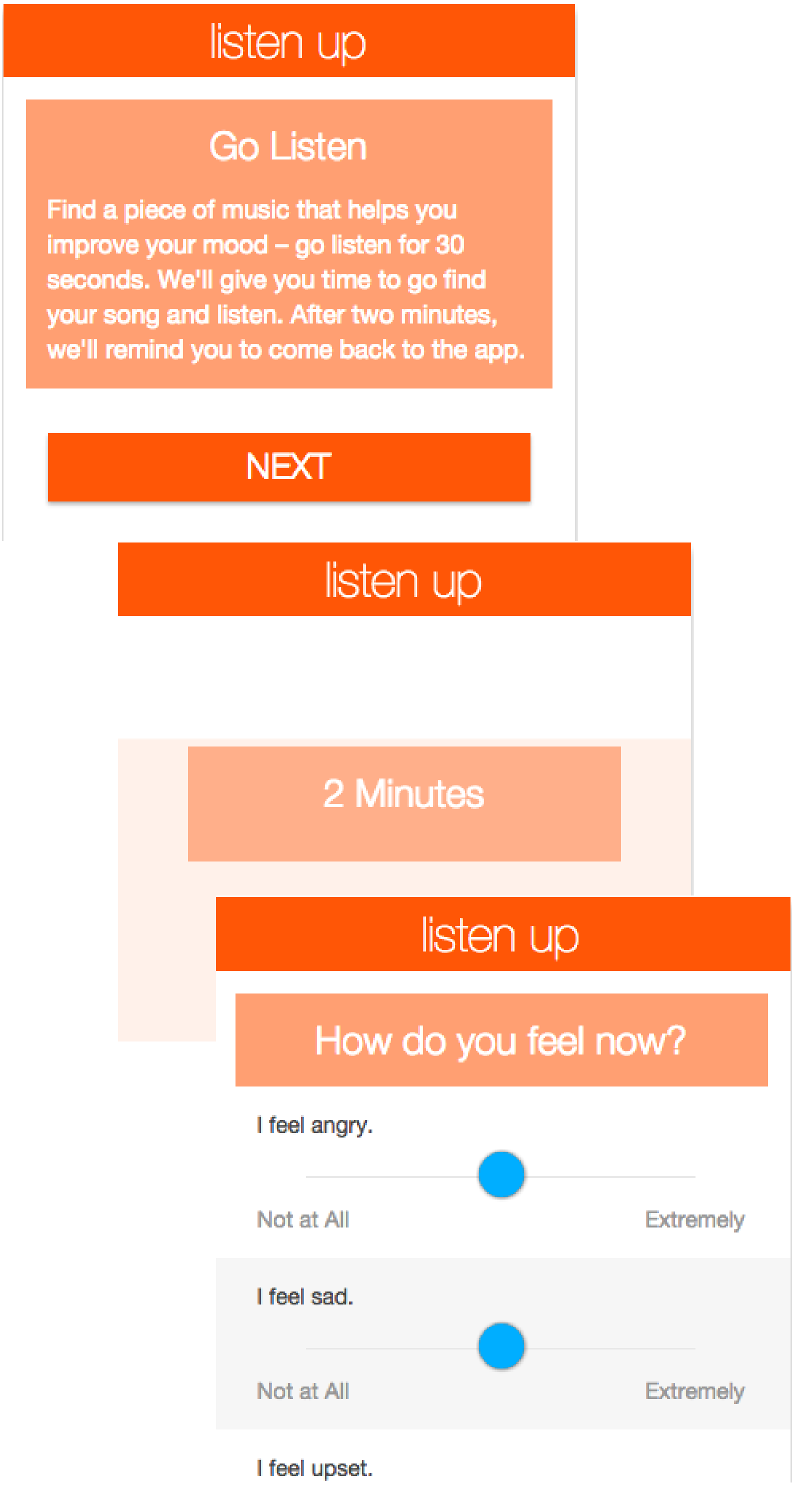

Push Notification Schedule for CharacterMe Activities

Participants were sent push notifications to engage in particular activities each day. They were also allowed to freely choose activities as they progressed through the app.

Day 1: Watch Get Better Framing Video; Emotion Thermometer

Day 2: Watch Solve a Conflict Framing Video; Math Challenge; Emotion Thermometer

Day 3: Mindfulness; Emotion Thermometer

Day 4: Watch Your Mouth; Selfie; Emotion Thermometer

Day 5: Listen Up; Take Perspective; Emotion Thermometer

Day 6: Hand Swap; Take Perspective; Emotion Thermometer

Day 7: Watch Your Mouth ; Solutions; Emotion Thermometer

Day 8: Solutions; Watch Get Better Framing Video; Emotion Thermometer

Day 9: Hand Swap; Solutions; Emotion Thermometer

Day 10: Watch Solve a Conflict Framing Video: Watch Your Mouth; Emotion Thermometer

Day 11: Think Again; Hand Swap; Emotion Thermometer

Day 12: Watch Your Mouth ; Think Again; Emotion Thermometer

Day 13: Think Again; Emotion Thermometer

Day 14: Math Challenge; Emotion Thermometer

3-Factor Patience Questionnaire

Instructions: For each of the statements below, please indicate how much the statement is like/unlike you.

1 = Not like me at all
2 = Unlike me
3 = Neutral
4 = Like me
5 = Very much like me

_____ 1. My friends would say I'm a very patient friend.
_____ 2. I am able to wait-out tough times.
_____ 3. Although they're annoying, I don't get too upset when stuck in a traffic jam.
_____ 4. I am patient with other people.
_____ 5. I find it pretty easy to be patient with a difficult life problem or illness.
_____ 6. In general waiting in lines does not bother me.
_____ 7. I have trouble being patient with my close friends and family.
_____ 8. I am patient during life hardships.
_____ 9. When someone is having difficulty learning something new, I will be able to help them without getting frustrated or annoyed.
_____ 10. I get very annoyed at red lights.
_____ 11. I find it easy to be patient with people.

Factor 1 – Interpersonal patience: 1, 4, 7(r), 9, 11
Factor 2 – Life hardships patience: 2, 5, 8
Factor 3 – Daily hassles patience: 3, 6, 10(r)

Self-control Scale (Tangney, Baumeister & Boone, 2004)

Tangney, J.P., Baumeister, R.F., & Boone, A.L. (2004). High self-control predicts good adjustment, less pathology, better grades and interpersonal success. *Journal of Personality*, *72*, 271-324.

Using the scale provided, please indicate how much each of the following statements reflects how you typically are.

			Not at all	Very much
	*	I am good at resisting temptation.	1— —2— —3— —4— —5	
(R)	*	I have a hard time breaking bad habits.	1— —2— —3— —4— —5	
(R)	*	I am lazy.	1— —2— —3— —4— —5	
(R)	*	I say inappropriate things.	1— —2— —3— —4— —5	
(R)	*	I do certain things that are bad for me, if they are fun.	1— —2— —3— —4— —5	
	*	I refuse things that are bad for me.	1— —2— —3— —4— —5	
(R)	*	I wish I had more self-discipline.	1— —2— —3— —4— —5	
	*	People would say that I have iron self- discipline.	1— —2— —3— —4— —5	
(R)	*	Pleasure and fun sometimes keep me from getting work done.	1— —2— —3— —4— —5	
(R)	*	I have trouble concentrating.	1— —2— —3— —4— —5	
	*	I am able to work effectively toward long-term goals.	1— —2— —3— —4— —5	
(R)	*	Sometimes I can't stop myself from doing something, even if I know it is wrong.	1— —2— —3— —4— —5	
(R)	*	I often act without thinking through all the alternatives.	1— —2— —3— —4— —5	
*	Items included in the Brief Self Control measure			
(R)	Reversed Items			

Children's Sadness and Anger Management

Directions: Reflecting on the past year, to what extent were the following statements true about ***you***:

**0**	**1**	**2**
Not True	Somewhat True	Very True

	**0**	**1**	**2**
1. When I am feeling sad, I control my crying and carrying on.	○	○	○
2. I stay calm and don't let sad things get to me.	○	○	○
3. I whine/fuss about what's making me sad.	○	○	○
4. When I'm sad, I do something totally different until I calm down.	○	○	○
5. I can stop myself from losing control over my sad feelings.	○	○	○
6. I cry and carry on when I'm sad.	○	○	○
7. I try to calmly deal with what is making me feel sad.	○	○	○
8. I do things like mope around when I'm sad.	○	○	○
9. When I'm feeling mad, I control my temper.	○	○	○
10. I stay calm and keep my cool when I'm feeling mad.	○	○	○
11. I do things like slam doors when I'm mad.	○	○	○
12. I attack whatever it is that makes me mad.	○	○	○
13. I can stop myself from losing my temper.	○	○	○
14. I say mean things to others when I'm mad.	○	○	○
15. I try to calmly deal with what is making me feel mad.	○	○	○

Measures Administered to Participants

Virtue Measures *– Virtue measures are conceptualized as the dependent variables that will differentially change as a result of the interventions and experimental conditions.*

1. Self-Control Scale. Tangney, Baumeister, & Boone's (2004) scale contains items measuring self-control (e.g., "I am good at resisting temptation") on a *1 (Not at All)* to *5 (Very Much)* scale. We will use the abbreviated, 13-item version of the scale. (Items included in the brief version are marked with an asterisk in Appendix B.)

2. 3-*Factor Patience Questionnaire.* The 11-item 3-Factor Patience Questionnaire (3-FPQ; Schnitker, 2012) will be used as a measure of interpersonal ("My friends would say I'm a very patient friend"), life hardships ("I find it pretty easy to be patient with a difficult life problem or illness"), and daily hassles ("Although they're annoying, I don't get too upset when stuck in a traffic jam") patience. Items are rated from 1 = *Not like me at all* to 5 = *Very Much Like Me*.

3. General Regulatory Behavior Questionnaire. A questionnaire was developed based on Oaten and Cheng's (2004) study items measuring everyday regulatory behaviors, including cigarette smoking, alcohol and caffeine consumptions, dietary habits, self-care habits, spending habits, emotion control, study habits, obeying rules, use of social media, energy conservation, and turning in assignments. Participants were asked to rate how often they engaged in behaviors in the past week on a scale from 9 (*not at all*) to 5 (*almost always*).

4. Emotion regulation of participants will be assessed using two subscales (i.e., 4-item emotion regulation coping and 3-item dysregulated expression) from the Children's Sadness and Anger Management Scale (Zeman, Shipman, & Penza-Clyve, 2001. Participants will report on sadness and anger. A sample item follows: "When I am feeling mad, I control my temper" (anger/sadness regulation coping), and "I attack whatever it is that makes me mad." (dysregulated coping). The response choices follow: 0 = "not true," 1 = "somewhat true," 2 = "very true". Participants will also complete Gross and John's (2003) Emotion Regulation Questionnaire (ERQ), which assesses individual differences in utilizing the two emotion regulation strategies of cognitive reappraisal (e.g., "I control my emotions by *changing the way I think* about the situation I'm in") and expressive suppression (e.g., "When I want to feel less negative emotion (such as sadness or anger), I *change what I'm thinking about*"). The 10 items are rated on a 7-point Likert scale.

Spirituality/Religiosity Measures *– Spirituality and religiosity measures are expected to moderate the effects of the interventions on virtue development. The measures are also included for manipulation check purposes.*

5. Duke University Religious Index (DUREL). The Duke University Religion Index (DUREL; Koenig & Bussing, 2010) is a five-item measure of religious involvement that includes dimensions of religiosity (organizational religious activity, non-organizational religious activity, and intrinsic religiosity). Organizational religious activity (ORA; How often do you attend church or other religious meetings?) and non-organizational religious activity (NORA; How often do you spend time in private religious activities, such as prayer, meditation or Bible?) are both rated on a 6 point scale scale. For ORA the response choice is as follows: 1 - Never; 2 - Once a year or less; 3 - A few times a year; 4 - A few times a month; 5 - Once a week; 6 - More than once/week; while, NORA includes: 1 - Rarely or never; 2 - A few times a month; 3 - Once a week; 4 - Two or more times/week; 5 - Daily; 6 - More than once a day. Intrinsic religiosity is measured based on three items (e.g. In my life, I experience the presence of the Divine (i.e., God) and the response choices are 1 - Definitely not true; 2 - Tends not to be true; 3 - Unsure; 4 - Tends to be true; 5 - Definitely true of me.

6. *God Concept Questions*. Based on Laurin, Kay, and Fitzsimons (2012), perceptions of God's sovereignty and watchfulness. Perceptions of God's sovereignty will be rated from 1 to 5 based on the degree to which the person conceives of God as in control of their success or failure in life. "My future success in life depends...(1) completely on factors God controls (2) mostly on factors that God controls (3) equally on factors that God and I control (4) mostly on factors that I control (5) completely on factors I control. Perceptions of God watching will be rated from 1 to 5 on a

Likert scale. “If God (or some non-human spiritual being) exists, it is likely that God watches peoples’ behavior and notices when they misbehave.” Additionally, the monitoring by God subscale (3 items) based on Carter, McCullough & Carver (2012) will also be used. Ratings will be based on a 1 (*Not at all*) to 7 (*Very true*) scale and include items such as “I believe a higher power can see my behavior.”

7. Perception of Perfectionism of God. The Perceived Perfectionism from God Scale (PPGS) was developed based on the Almost Perfect Scale-Revised (APS-R). Ratings will be made on a 1 (*Strongly Disagree*) to 7 (*Strongly Agree*) scale. It consists of two core perfectionism dimensions (Standards, 6-items & Discrepancy, 9-items). Standards are the adaptive aspect with items like “God expects the best from me”, whereas discrepancy (the gap between their standards and performance) is the negative aspect and includes items such as “My performance rarely measures up to God's standards”.

8. Attitudes Toward God Scale-9 (Wood et al., 2010) assesses the extent to which participants currently feel anger/disappointment toward God with four items (e.g., “Feel that God has let you down”) and comfort from God with five items (e.g., “Feel loved by God”). Items were rated from 1 = not at all to 10 = extremely.

***Well-Being Measures** – In addition to expecting increases in virtue as a result of our interventions and experimental conditions, we expect that well-being will also increase. Thus, these will be used as dependent variables.*

9. Satisfaction with Life Scale. This 5-item scale (Diener, Emmons, Larsen, & Griffin, 1985) measures global satisfaction with life. Items such as “In most ways, my life is close to my ideal” are rated on a scale from 1 *(strongly disagree)* to 7 *(strongly agree).*

10. Positive and Negative Affect Schedule. The shortened version of the PANAS-C (Ebesutani et al., 2012; Laurent, et al. 1999) will be used that includes 5-item PA scale (joyful, cheerful, happy, lively, proud) and a 5-item NA scale (miserable, mad, afraid, scared, sad) rated on a 1 (*very slightly or not at all*) to 5 (*extremely*) scale.

11. CES-D. To assess depression, a 10-item revised version (Kohout, Berkman, Evans, & Cornoni-Huntley, 1993) of the longer 20-item measure from the Center for Epidemiologic Study’s Inventory for depressed mood (CES-D; Radloff, 1977) will be used. Participants will be asked how often they felt a particular way during the past week. The following item is from the measure: “I felt that everything I did took a lot of effort.” From the 10 items on the scale, two will be reversed-coded (e.g., “I was happy” and “I enjoyed life”). Response choices follow: 0 = rarely or none of the time (less than 1 day), 1 = some or a little of the time (1-2 days), 2 = occasionally (3-4 days), 3 = mostly or almost all the time (5-7 days). Cutoff scores for screening and further review by a clinician are set at an average score of 3.5. For detailed information regarding the screening process activated by a high score, see section VII – Potential Risks below.

12. The 7-item *Generalized Anxiety Disorder* scale (GAD-7) will be used to assess adolescents’ generalized anxiety (Spitzer, Kroenke, Williams, & Löwe, 2006). The stem for the scale follows: “Over the last two weeks, how often have you been bothered by the following problems.” A sample item follows: “Worrying too much about different things.” The response choices follow: 1 = not at all, 2 = several days, 3 = more than half the days, and 4 = nearly every day.

13. UCLA Loneliness Scale. The ULS-3 (Hays & DiMatteo, 1987; c.f. Russell, Peplau, & Cutrona, 1980) is a 3-item scale designed to measure loneliness. Items include “I lack companionship” and “I feel left out”. These items are rated on a 4-point Likert scale from 1 (*Never*) to 4 (*Often*).

14. Youth Risk Behavior Surveillance. Participants will be administered 16 items selected from Youth Risk Behavior Surveillance measure utilized by Center for Disease control (Foti, Balaji, & Shanklin, 2011). Items to be administered relate to tobacco, alcohol, and marijuana use, dietary habits, exercise activities, safe driving, and media/technology use.

26. Meaning in Life Questionnaire. The 5-item Presence of Meaning subscale from the Meaning in Life Questionnaire (Strenger, Frazier, Oishi, & Kaler, 2006) will be used to assess purpose and

meaning in life. Items such as “My life has a clear sense of purpose” are rated on a 7-point likert scale (1 = Absolutely Untrue to 7 = Absolutely True).
Personality Measures *– To be used as control variables (Big Five), manipulation checks (ERQ), or moderators of the interventions effectiveness (self-efficacy).*
15. Big Five Inventory. The 10-item BFI (Gosling, Rentfrow, & Swann, 2003; Rammstedt & John, 2007) measures the personality factors of extraversion (e.g., “I see myself as someone who is reserved”), agreeableness (e.g., “I see myself as someone who is generally trusting”), conscientiousness (e.g., “I see myself as someone who does a thorough job”), neuroticism (e.g., “I see myself as someone who gets nervous easily”), and openness to experience (e.g., “I see myself as someone who has an active imagination”) on a *1 = Disagree Strongly* to *5 = Agree Strongly* Likert scale.
16. Self-Efficacy Scale. The Self-Efficacy Scale (Sherer et al., 1982) is a 23-item scale that measures personal mastery. It has two subscales, a general self-efficacy scale (“When I make plans, I am certain I can make them work), and a social self-efficacy scale (“If I see someone I would like to meet, I go to that person instead of waiting for him or her to come to me”). The items are rated on a 14-point Likert scale from 1 (*strongly disagree*) to 14 (*strongly agree*).
17. *Implicit Theories of Morality and Talent Measures.* Dweck, Chiu, & Hong’s (1995) Implicit Theories of Morality measure contains items measuring an entity view of morality (e.g., "A person's moral character is something very basic about them and it can’t be changed much”). The implicit theories of morality measure consists of 3 items. Participants will be asked if they agree with the item on a 6-point scale, from *1 (Very Strongly Agree)* to *6 (Very Strongly Disagree).*) The theories of talent measure contains items measuring an entity view of talent. The theories of intelligence measure consists of 3 items, each depicting intelligence as a fixed entity (e.g., "You have a certain amount of talent and you really can't do much to change it"). Participants will be asked if they agree with the item on a 6-point scale, from *1 (Very Strongly Agree)* to *6 (Very Strongly Disagree).*
18. *Moral, Spiritual, and Instrumental Motives*. Three items will be used to assess the importance of goals related to improving performance/achievement, spiritual growth, and moral character on a 1 (*strongly disagree*) to 5 (*strongly agree*) scale.
Items Related to Athletic/Extracurricular Activity and Social Support *– These measures will be used as outcomes of the intervention (self-reported performance) or moderators of the intervention efficacy (entitativity and coach measures).*
19. Group Entitativity Measure. The GEM-in (Gaertner & Schopler, 1998) measures perceived interconnections between self and others. Test-takers choose between 6 different pictures of the self and others (pictorially represented by circles). The first picture has the greatest distance between self and others, while the last picture shows no distance between self and others, so that the group and self are completely overlapping. Participants will be instructed to complete the measure relative to their athletic teams/extracurricular activity group (alternative instructions).
20. Self-Report Athletic/Extra-curricular Commitment, Effort, and Performance. A simple three-item self-report measure will be included in to measure athletic performance. Participants are asked to rate on a 7-point Likert Scale their athletic performance over the past week (1 = Strongly Disagree, 7 = Strongly Agree). Statements will include: “During practice, I was able to prepare for upcoming games, competitions, or other events to increase my athletic performance,” “I feel I performed at the height of my ability during sporting events this last week (e.g., games, scrimmages, competitions),” and “My athletic performance, when measured as much as possible by stats (e.g., points scored, assists, times) was higher last week than it has been lately.”

A similar three-item self-report measure will be included to assess performance amongst non-athletes. Participants are asked to rate on a 7-point Likert Scale their performance in extracurricular or other activities over the past week (1 = Strongly Disagree, 7 = Strongly Agree). Statements will include: “During practice (or rehearsal), I was able to prepare for performances, upcoming events, competitions, etc.”, “I feel I performed at the height of my ability during

performances, practices, events, and competitions this past week (e.g., games, scrimmages, competitions)", "My performance, when measured by other people (e.g., instructors, peers), was higher last week than it has been lately."

Two additional items will assess commitment and effort on for all participants. The first item will ask participants to "Please rate your level of commitment to your sport/extracurricular activity" on a 1 = Very Low Commitment to 7 = Very High Commitment scale. The second item will ask, "How much effort have you exerted in relation to your sport/extracurricular activity in the past week?" on a 1 = Very Low Effort to 7 = Very High Effort scale.

21. Coach/Extracurricular Instructor Ratings. Participants will complete the 16-item Perceived Available Support in Sport Questionnaire (PASS-Q; Freeman, Coffee, & Rees, 2011), which measures perceived emotional, esteem, information, and tangible support. The items are preceded by a generic stem that asked, "If needed, to what extent would someone . . . , " with participants responding on a 5-point Likert scale ranging from 0 *(not at all)* to 4 *(extremely so)*. Higher values represented higher levels of perceived available support. Instructions will be adjusted to the most important extracurricular activity for non-athletes. In addition, participants will complete 10 items from Shields, LaVoi, Bredemeier, & Power (2007) on perceived poor sportspersonship behavior of the coach. For example, participants will rate how often coaches encouraged cheating or aggression in the season on a scale from *1 (never)* to *4 (often)*.

*22. Performance Failure Appraisal Inventory***.** Participants will be administered a short-form measure of general fear of failure using 5 items (Conroy, Poczwardowski, & Henschen, 2001). Items on this measure include "When I am not succeeding, people are less interested in me" and "When I am failing, it upsets important others".

23. Contingencies of Self-worth. Further, participants will report on 3 subscales of contingencies of self-worth rated on a 1 (Strongly disagree) to 7 (Strongly agree) and each subscale has 5 items (Crocker et al., 2003; competition, virtue, God's love). Participants will rate Competition contingencies will include statements such as "my self-worth is affected by how well I do when I am competing with others". Virtue contingencies includes statements like "I couldn't respect myself if I didn't live up to a moral code" and "When I think that I am disobeying God, I feel bad about myself" for God's love contingencies.

24. The 4-item parental support scale assessed perceived behaviors from mothers and fathers that communicate to the participant feelings of warmth, affection, and a sense of being valued and the 7-item parental monitoring scale assessed parental monitoring (Bush, Peterson, Cobas, & Supple, 2002; Henry & Peterson, 1995; Peterson, Rollins, & Thomas, 1985). Participants were asked to respond to each item twice: once about their primary mother figure and once about their primary father figure. A sample item follows: "This parent seems to approve of me and the things I do." Response choices follow: 1 = *strongly disagree*, 2 =*disagree*, 3 = *agree*, 4 = *strongly agree*. The items were averaged to create scores for each parent.

25. Demographics

Assessments Made During Intervention Activities **–** *Will be used as moderators of the intervention efficacy.*

26. Ratings of intervention enjoyability, success, motivation, and difficulty. Each day that participants record their progress on their intervention activity, they will rate the enjoyability, success, motivation, and difficulty for the activity. This rating will apart of the survey they will receive daily and they will have an opportunity to reflect on their experience of the activities (i.e. *Rate how well you enjoyed today's activity).*

Objective Indicators of Athletic Performance

27. Objective Indicators of Athletic Performance. Various sport-dependent objective indicators of athletic performance and effort will be collected directly from coaches.

Measures Administered to Coaches/Activity Advisors

Self-Reports of:

1. Self-Control Scale. Tangney, Baumeister, & Boone's (2004) – see description above.
2. 3-*Factor Patience Questionnaire.* 3-Factor Patience Questionnaire (Schnitker, 2012) – see description above.
3. *Implicit Theories of Morality and Talent Measures.* Dweck, Chiu, & Hong's (1995) Implicit Theories of Morality measure – see description above.
Reports of a particular participant's:
1. Self-Control Scale. Tangney, Baumeister, & Boone's (2004) – see description above.
2. 3-*Factor Patience Questionnaire.* – see description above.
3. General Regulatory Behavior Questionnaire. – see description above.
4. Satisfaction with Life Scale. Diener, Emmons, Larsen, & Griffin (1985) – see description above.
5. *Emotion regulation* – Children's Sadness and Anger Management Scale (Zeman, Shipman, & Penza-Clyve, 2001). – see description above.
6. Participant's Athletic/Extra-curricular Commitment, Effort, and Performance. – see description above.
26. *Meaning in Life Questionnaire*. Strenger, Frazier, Oishi, & Kaler, 2006 – see description above

<u>Measures Administered to Peer Informants</u>
Self-Reports of:
1. Self-Control Scale. Tangney, Baumeister, & Boone's (2004) – see description above.
2. 3-*Factor Patience Questionnaire.* 3-Factor Patience Questionnaire (Schnitker, 2012) – see description above.
3. General Regulatory Behavior Questionnaire. Oaten and Cheng (2004) – see description above.
4. *Emotion regulation* – Children's Sadness and Anger Management Scale (Zeman, Shipman, & Penza-Clyve, 2001). – see description above.
Reports of a particular participant's:
1. Self-Control Scale. Tangney, Baumeister, & Boone's (2004) – see description above.
2. 3-*Factor Patience Questionnaire.* – see description above.
3. General Regulatory Behavior Questionnaire. – see description above.
4. *Emotion regulation* – Children's Sadness and Anger Management Scale (Zeman, Shipman, & Penza-Clyve, 2001). – see description above.
5. Satisfaction with Life Scale. Diener, Emmons, Larsen, & Griffin (1985) – see description above.
26. *Meaning in Life Questionnaire*. Strenger, Frazier, Oishi, & Kaler, 2006 – see description above

<u>Study 1 Measures Administered to Parents</u>
Self-reports of:
1. Self-Control Scale. Tangney, Baumeister, & Boone's (2004) – see description above.
2. 3-*Factor Patience Questionnaire.* 3-Factor Patience Questionnaire (Schnitker, 2012) – see description above.
3. General Regulatory Behavior Questionnaire. Oaten and Cheng (2004) – see description above.
4. Perception of Perfectionism of God.
5. DUREL. See above
6. *God Concept Questions*. Based on Laurin, Kay, and Fitzsimons (2012) and Carter, McCullough & Carver (2012) – see description above.
7. *Implicit Theories of Morality and Talent Measures.* Dweck, Chiu, & Hong's (1995) Implicit Theories of Morality measure – see description above.
Reports of their child's:
1. Self-Control Scale. Tangney, Baumeister, & Boone's (2004) – see description above.

2. 3-*Factor Patience Questionnaire*. – see description above.
3. General Regulatory Behavior Questionnaire. – see description above.
4. Satisfaction with Life Scale. Diener, Emmons, Larsen, & Griffin (1985) – see description above.
5. *Emotion regulation* – Children's Sadness and Anger Management Scale (Zeman, Shipman, & Penza-Clyve, 2001). – see description above.
6. Participant's Athletic/Extra-curricular Commitment, Effort, and Performance. – see description above.
26. *Meaning in Life Questionnaire*. Strenger, Frazier, Oishi, & Kaler, 2006 – see description above

App Framing Error Details

When we cleaned the data for analyses, we discovered some errors in the framing manipulation language viewed by the participants in the Thermometer, Hand Swap, and Watch Your Mouth activities. Below is a fully accounting of the content received by participants in each condition. ***(Italicized bolded text indicates errors.)***

Instrumental/Avoidance

- Personal Reflection Task: Instrumental/Avoidance
- Get Better Framing Video (2xs): Instrumental/Avoidance
- Solve a Conflict Framing Video (2xs): Instrumental/Avoidance
- Thermometer: ***Moral/Approach***
- Hand Swap: ***Moral/Approach***
- Watch Your Mouth: ***Moral/Approach***

Instrumental/Approach

- Personal Reflection Task: Instrumental/Approach
- Get Better Framing Video (2xs): Instrumental/Approach
- Solve a Conflict Framing Video (2xs): Instrumental/Approach
- Thermometer: Instrumental/Approach
- Hand Swap: Instrumental/Approach
- Watch Your Mouth: Instrumental/Approach

Moral/Avoidance

- Personal Reflection Task: Moral/Avoidance
- Get Better Framing Video (2xs): Moral/Avoidance
- Solve a Conflict Framing Video (2xs): Moral/Avoidance
- Thermometer: ***Instrumental/Avoidance***
- Hand Swap: ***Instrumental/Avoidance***
- Watch Your Mouth: ***Instrumental/Avoidance***

Moral/Approach

- Personal Reflection Task: Moral/Approach
- Get Better Framing Video (2xs): Moral/Approach
- Solve a Conflict Framing Video (2xs): Moral/Approach
- Thermometer: ***Spiritual/Approach***
- Hand Swap: ***Spiritual/Approach***
- Watch Your Mouth: ***Spiritual/Approach***

Spiritual/Avoidance

- Personal Reflection Task: Spiritual/Avoidance
- Get Better Framing Video (2xs): Spiritual/Avoidance
- Solve a Conflict Framing Video (2xs): Spiritual/Avoidance
- Thermometer: Spiritual/Avoidance
- Hand Swap: Spiritual/Avoidance
- Watch Your Mouth: Spiritual/Avoidance

Spiritual/Approach

- Personal Reflection Task: Spiritual/Approach
- Get Better Framing Video (2xs): Spiritual/Approach
- Solve a Conflict Framing Video (2xs): Spiritual/Approach
- Thermometer: ***Moral/Approach***
- Hand Swap: ***Moral/Approach***
- Watch Your Mouth: ***Moral/Approach***

Supplemental Table 1

Condition Specific Framing Language for Thermometer, Hand Swap, and Watch your Mouth

Thermometer	**instrumental**	**moral**	**spiritual**
**positive**	Healthy people pay attention to how they feel.	People who make good choices pay attention to how they feel	People who are connected to a higher power are in touch with how they feel
**negative**	Unhealthy people ignore how they feel.	People who don't pay attention to how they feel don't make good choices	People who are not connected to how they feel are disconnected from a higher power

Hand Swap	**instrumental**	**moral**	**spiritual**
**positive**	Using your non-dominant hand for everyday tasks builds self-control.	Using your non-dominant hand increases self-control and can help you to choose the right thing to do in tough situation	Using your non-dominant hand increases the virtue of self-control making you more connected to God or a higher power
**negative**	Only using your dominant hand makes you weak.	Only using your dominant hand makes you to less likely to choose the right thing to do in tough situation	People who only use their dominant hand lack self-control and are less connected to a higher power

Watch Your Mouth	**instrumental**	**moral**	**spiritual**
**positive**	Controlling the words you use builds self-control.	Controlling the words you use can make you more fair and just	Controlling the words you use can make you more connected to God or a higher power
**negative**	Not controlling the words you use makes you weak	Not controlling the words you use can lead you to be less fair and just	Not controlling the words you use can disconnect you from God or a higher power

Supplemental Analyses with Religiosity as Covariate

We ran exploratory LGCMs controlling for religiosity to assess whether the participants' initial levels of religiosity would affect the spiritual framing condition effects.

Religiosity was assessed using the intrinsic religiosity scale of the Duke University Religion Index (DUREL; Koenig & Bussing, 2010), which included three items (e.g., "In my life, I experience the presence of the Divine (i.e., God)"), and the response choices ranged from 1 = *definitely not true* to 5 = *definitely true of me*. Additionally, participants completed the Attitudes Toward God Scale-9 (Wood et al., 2010), which assessed the extent to which participants feel anger/disappointment toward God with four items (e.g., "Feel that God has let you down") and comfort from God with five items (e.g., "Feel loved by God"). Items were rated from 1 = not at all to 10 = *extremely*. Internal reliability was .93 for intrinsic religiosity, .87 for anger toward God, and .98 for comfort from God. Both the DUREL (e.g., Helms et al., 2015) and Attitudes Toward God-9 (Park & Cho, 2016) have been used with adolescents to predict well-being across time.

Results are shown in Supplemental Tables 2a and 2b. When we ran the conditional models with the inclusion of intrinsic religiosity, comfort from God, and anger toward God as covariates, there were only minor changes in the pattern of findings. As seen in Supplemental Table 2b, all findings that were previously significant without controlling for religiosity remained significant and effect sizes similar. There were two new significant findings for anger regulation. Greater engagement with the Watch Your Mouth challenge was associated with a positive change in anger regulation, but the effect was very small with a coefficient < .001. In comparison to the instrumental/avoidance framing, the moral/avoidance framing predicted more positive within-person change in anger regulation in addition to life hardships and interpersonal patience. However, the religiosity covariates were not statistically significant predictors in the conditional LGCM for anger regulation, so these additional results should be interpreted with caution.

Helms, S. W., Gallagher, M., Calhoun, C. D., Choukas-Bradley, S., Dawson, G. C., & Prinstein, M. J. (2015). Intrinsic religiosity buffers the longitudinal effects of peer victimization on adolescent depressive symptoms. *Journal of Clinical Child & Adolescent Psychology*, *44*(3), 471-479.

Koenig, H. G., & Büssing, A. (2010). The Duke University Religion Index (DUREL): A five-item measure for use in epidemiological studies. *Religions*, *1*(1), 78-85.

Park, C. L., & Cho, D. (2017). Spiritual well-being and spiritual distress predict adjustment in adolescent and young adult cancer survivors. *Psycho-oncology, 26*(9), 1293-1300.

Wood, B. T., Worthington Jr, E. L., Exline, J. J., Yali, A. M., Aten, J. D., & McMinn, M. R. (2010). Development, refinement, and psychometric properties of the Attitudes Toward God Scale (ATGS-9). *Psychology of Religion and Spirituality, 2*(3), 148.

Supplemental Table 2a and 2b

Table 2a

Model Fit Indices for Latent Growth Curve Models

	AIC	BIC	$\chi^2(df)$	CFI	TLI	RMSEA
**Conditional Model Controlling for Religiosity**						
Life Hardships Patience	3104.33	3241.86	83.70*	0.96	0.94	0.03
Interpersonal Patience	2412.21	2549.74	81.94*	0.97	0.96	0.03
Daily Hassles Patience	3005.83	3143.36	102.76***	0.93	0.90	0.04
Self-Control	1459.46	1596.92	72.85	0.98	0.97	0.02
Anger Regulation	1612.24	1753.94	68.79	0.98	0.97	0.02
Sadness Regulation	1255.99	1393.52	92.49	0.92	0.88	0.03

Note. AIC = Akaike Information Criteria, BIC = Bayesian Information Criterion; RMSEA = Root Mean Square Error of Approximation, CFI = Comparative Fit Index. **p* <. 05 ***p* < .01 ****p* < .001

Table 2b

Parameter Estimates for LGC Models Controlling for Religiosity

	Life Hardships Patience	Inter-personal Patience	Daily Hassles Patience	Self-Control	Anger Regulation	Sadness Regulation
**Conditional Model Controlling for Religiosity**						
Gender	-0.006	-0.004	0.001	0.000	-0.007	-0.001
Age	-0.002	-0.001	-0.003	-0.002	-0.001	0.001
Intrinsic Religiosity	-0.094	0.000	-0.011**	0.002	0.001	0.002
Comfort from God	-0.144	-0.001	-0.003*	0.000	0.001	0.002
Anger toward God	0.131*	0.001	0.001	0.002*	-0.001	-0.001
Hand Swap	-0.001***	0.000	-0.001	0.000	0.000	0.001*
Math	-0.001*	-0.001	0.000	-0.001	0.000	0.000
Watch Your Mouth	0.001*	0.000	0.000	0.000	0.000*	0.000*
Listen Up	-0.002	-0.001	-0.001	0.000	0.000	0.001
Mindfulness	-0.003	-0.004*	-0.002	0.000	-0.001	-0.001*
Selfie	0.001	0.000	-0.003	-0.001	0.001	0.002
Solutions	-0.001	0.000	0.000	0.000	0.000	0.001
Take Perspective	0.002**	0.001*	0.001	0.000	-0.001	-0.001*
Think Again	0.000	0.004*	0.002	-0.001	0.001	0.000
Total engagement	0.003*	0.000	0.000	0.000	-0.001	-0.002**
Framing						
Instrumental/Approach[A]	0.009	0.006	-0.013	0.001	0.010	0.004
Moral/Avoid[A]	0.027*	0.019**	-0.002	-0.004	0.018*	0.009
Moral/Approach[A]	0.018	0.005	0.002	-0.002	0.005	-0.002
Spiritual/Avoid[A]	-0.007	-0.002	-0.019	-0.014	-0.001	0.002
Spiritual/Approach[A]	0.002	0.014*	-0.012	0.002	0.010	0.004

Note. [A]Instrumental/Avoid framing is the reference group. Religion variables being controlled for are intrinsic religiosity from the DUREL (Koenig & Büssing, 2010) and comfort and anger toward God from the Attitudes toward God Scale (Wood et al., 2009).

**p* <. 05 ***p* < .01 ****p* < .001
